# SAGA1 and MITH1 produce matrix-traversing membranes in the CO_2_-fixing pyrenoid

**DOI:** 10.1038/s41477-024-01847-0

**Published:** 2024-11-15

**Authors:** Jessica H. Hennacy, Nicky Atkinson, Angelo Kayser-Browne, Sabrina L. Ergun, Eric Franklin, Lianyong Wang, Simona Eicke, Yana Kazachkova, Moshe Kafri, Friedrich Fauser, Josep Vilarrasa-Blasi, Robert E. Jinkerson, Samuel C. Zeeman, Alistair J. McCormick, Martin C. Jonikas

**Affiliations:** 1 Department of Molecular Biology, Princeton University, Princeton, NJ USA; 2 Institute of Molecular Plant Sciences, School of Biological Sciences, University of Edinburgh, Edinburgh, Scotland UK; 3 Howard Hughes Medical Institute, Princeton University, Princeton, NJ USA; 4 Department of Biology, ETH Zurich, Zurich, Switzerland; 5 Department of Plant Biology, Carnegie Institution for Science, Stanford, CA USA; 6 Department of Botany and Plant Sciences, University of California, Riverside, CA USA; 7 Department of Chemical and Environmental Engineering, University of California, Riverside, CA USA

**Keywords:** Cell biology, Photosynthesis

## Abstract

Approximately one-third of global CO_2_ assimilation is performed by the pyrenoid, a liquid-like organelle found in most algae and some plants. Specialized pyrenoid-traversing membranes are hypothesized to drive CO_2_ assimilation in the pyrenoid by delivering concentrated CO_2_, but how these membranes are made to traverse the pyrenoid matrix remains unknown. Here we show that proteins SAGA1 and MITH1 cause membranes to traverse the pyrenoid matrix in the model alga *Chlamydomonas reinhardtii*. Mutants deficient in *SAGA1* or *MITH1* lack matrix-traversing membranes and exhibit growth defects under CO_2_-limiting conditions. Expression of SAGA1 and MITH1 together in a heterologous system, the model plant *Arabidopsis thaliana*, produces matrix-traversing membranes. Both proteins localize to matrix-traversing membranes. SAGA1 binds to the major matrix component, Rubisco, and is necessary to initiate matrix-traversing membranes. MITH1 binds to SAGA1 and is necessary for extension of membranes through the matrix. Our data suggest that SAGA1 and MITH1 cause membranes to traverse the matrix by creating an adhesive interaction between the membrane and matrix. Our study identifies and characterizes key factors in the biogenesis of pyrenoid matrix-traversing membranes, demonstrates the importance of these membranes to pyrenoid function and marks a key milestone toward pyrenoid engineering into crops for improving yields.

## Abstract (web summary)

Two algal proteins, MITH1 and SAGA1, play key roles in formation of membranes that deliver CO_2_ to the pyrenoid, a CO_2_-concentrating organelle. Their discovery marks a key milestone towards engineering a pyrenoid into land plants for improved yields.

## Main

**Fig. 1:**
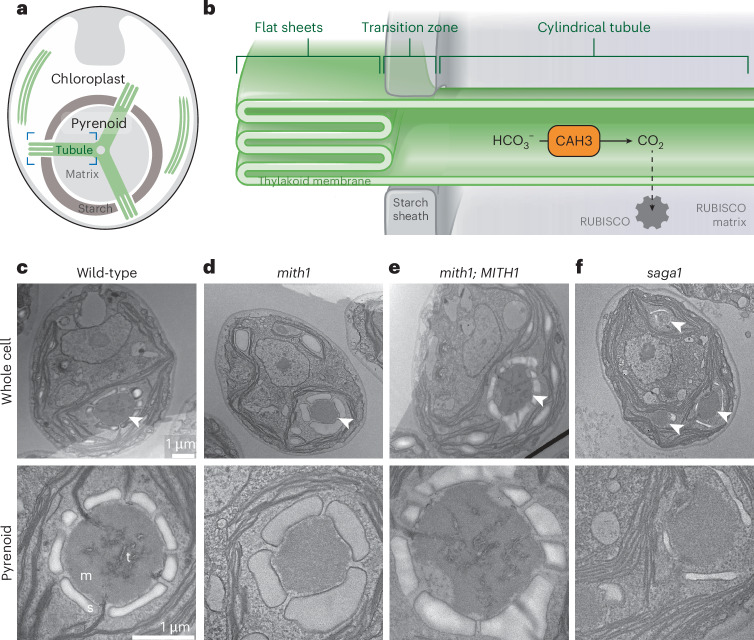
MITH1 and SAGA1 are necessary for pyrenoid matrix-traversing membranes in *Chlamydomonas.* **a**, The *Chlamydomonas* pyrenoid is found at the base of the cell’s cup-shaped chloroplast and consists of a Rubisco matrix traversed by membrane tubules that join in a reticulated region at the centre^12^. The pyrenoid is surrounded by a starch sheath thought to act as a barrier against CO_2_ escape^31^. **b**, Flat sheets of thylakoid membrane transition into cylindrical tubules as they enter the pyrenoid matrix through gaps in the starch sheath, according to cryo-electron tomography data^12^. Bicarbonate (HCO_3_^−^) is thought to be concentrated in the lumen of pyrenoid matrix-traversing membranes and converted to CO_2_ by carbonic anhydrase 3 (CAH3) (ref. ^22^). **c**–**f**, TEM images of whole *Chlamydomonas* cells and pyrenoids. The strains include (**c**) the wild-type CMJ030 (ref. ^46^), (**d**) a *mith1* mutant^36^, (**e**) a complemented strain generated by transforming MITH1-Venus into the *mith1* mutant background (see Methods) and (**f**) a *saga1* mutant^13^. Representative images from 3 biological repeats are shown. Arrowheads point to pyrenoids. t, traversing membrane tubules; m, matrix; s, starch. Images in each row are presented at the same scale.

The growth of photosynthetic organisms is commonly limited by the rate of CO_2_ fixation catalysed by the enzyme Rubisco^1^. Nearly all eukaryotic algae and some plants overcome this limitation with a chloroplast-localized organelle called the pyrenoid, which enhances CO_2_ fixation (Fig. 1a)^2^. The prevalence of pyrenoids in eukaryotic algae has led to the estimate that pyrenoids mediate 61–88% of primary productivity in the oceans^3^, making the pyrenoid an organelle of major biogeochemical importance. Furthermore, engineering a pyrenoid into major C3 crops such as wheat and rice is expected to enhance their CO_2_ uptake, yield and water use efficiency^4^, which will promote agricultural resilience in the face of climate change^5^.

Pyrenoids enhance CO_2_ fixation by supplying Rubisco with concentrated CO_2_. Almost all known pyrenoids share two ultrastructural features: a protein matrix and specialized membranes (Fig. 1a)^2^. The matrix is the site of CO_2_ fixation by Rubisco, and its biogenesis has recently been elucidated in the model alga *Chlamydomonas reinhardtii* (Chlamydomonas hereafter); it is a spheroidal liquid-like condensate^6^ composed primarily of Rubisco and an intrinsically disordered repeat linker protein, EPYC1 (refs. ^3,7^). EPYC1 and a compatible Rubisco were sufficient to reconstitute a matrix-like condensate in vitro^8^ and in the plant *Arabidopsis thaliana* (Arabidopsis hereafter), which does not natively contain a pyrenoid^9^.

The pyrenoid’s second ultrastructural feature consists of specialized membranes that traverse the matrix and have been hypothesized to perform the essential function of supplying concentrated CO_2_ to Rubisco (Fig. 1b)^10^. By contrast with the matrix, the biogenesis and function of these membranes have not been experimentally characterized in any organism. More broadly, matrix-traversing membranes represent an organellar configuration whose biogenesis has not yet been characterized at a molecular level in any system, where membranes traverse a phase-separated condensate. In the present work, we seek to address a basic question: which factors cause membranes to traverse the pyrenoid matrix?

## *mith1* and *saga1* mutants lack matrix-traversing membranes

**Extended Data Fig. 1:**
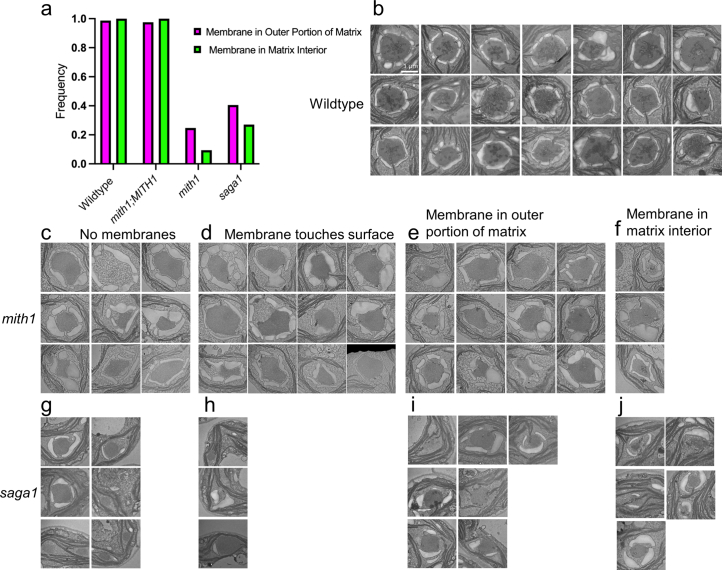
By TEM, *mith1* and *saga1* mutant pyrenoids are missing the canonical matrix-traversing membranes observed in wild-type pyrenoids. **a**, Graph depicting for each strain the percentage of pyrenoids with membranes seen entering the matrix surface or interior. n = 78 pyrenoids. **b**, TEM images of wild-type pyrenoids. **c-j**, TEM images of *mith1* and *saga1* mutant pyrenoids, organized into categories based on membrane contact with the pyrenoid. The ‘No membranes’ (**c, g**) category indicates that no membranes are seen in the pyrenoid. The ‘Membrane touches surface’ (**d, h**) category indicates that membranes are seen touching the surface of the pyrenoid, but are not seen entering the pyrenoid matrix. The ‘Membrane in outer portion of matrix’ category (**e, i**) describes pyrenoids where membranes are seen in the outer portion of the pyrenoid matrix. The ‘Membrane in matrix interior’ category (**f, j**) includes pyrenoids where membrane is seen at the interior of the matrix. All images are presented at the same scale as 1(b).

A high-throughput mutant screen in Chlamydomonas recently identified *MITH1* (*MIssing THylakoids 1, Cre06.g259100*, *SAGA3*) as a gene required for normal growth in low CO_2_, a characteristic of pyrenoid-related genes^11^. In the present study, using transmission electron microscopy (TEM), we found that *mith1* mutants lacked normal matrix-traversing membranes (Fig. 1 and Extended Data Fig. 1). In wild-type Chlamydomonas, matrix-traversing membranes appear as cylindrical tubules that merge with the photosynthetic thylakoid membrane sheets outside the pyrenoid^12^ (Fig. 1a–c). In the *mith1* mutant, matrix-traversing membranes were almost never observed (Fig. 1d and Extended Data Fig. 1). To our knowledge, this is presumably the first observation of a mutant with a phenotype specifically affecting the matrix-traversing membranes. In the complemented strain *mith1*;*MITH1-Venus*, an apparently normal matrix-traversing membrane tubule network is restored (Fig. 1e), demonstrating that MITH1 is necessary for the formation of matrix-traversing membranes.

The discovery of a role for MITH1 in matrix-traversing membrane biogenesis suggested a similar function for its homologue, SAGA1 (ref. ^11^) (Cre11.g467712), a protein that is also required for growth under low CO_2_ but whose function had remained elusive due to the *saga1* mutant’s complex phenotypes that impacted multiple pyrenoid subcompartments^13^. The idea that SAGA1 functions in the same matrix-traversing membrane biogenesis pathway as MITH1 is supported by the observation that *saga1* and *mith1* mutants displayed a similar pattern of growth defects across 121 different conditions in a high-throughput study^11^. Further consistent with a possible function for SAGA1 in producing matrix-traversing membranes, the *saga1* mutant exhibited a similar lack of matrix-traversing membranes to *mith1* (ref. ^13^) (Fig. 1f and Extended Data Fig. 1).

## MITH1 and SAGA1 produce matrix-traversing membranes in plants

**Fig. 2:**
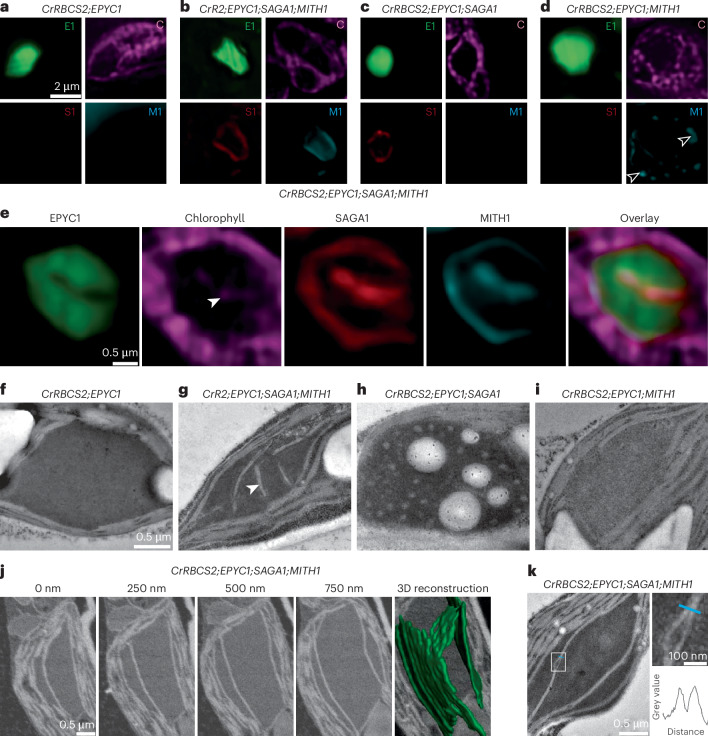
MITH1 and SAGA1 are sufficient to cause membranes to traverse the Rubisco matrix in Arabidopsis*.* **a**–**d**, Fluorescence images depicting Arabidopsis lines expressing different combinations of Chlamydomonas proteins. Images are presented at the same scale. E1, EPYC1-GFP; C, chlorophyll; S1, SAGA1-mCherry; M1, MITH1-mCerulean; CrR2, CrRBCS2, the Chlamydomonas Rubisco small subunit. Arrowheads in **d** point to MITH1 signal found outside the EPYC1-GFP condensate. **e**, High-resolution fluorescence images of a condensate found in the *CrRBCS2;EPYC1-GFP;SAGA1-mCherry;MITH1-mCerulean* line. Arrowhead points to matrix-traversing chlorophyll signal. **f**–**i**, TEM images of CrRBCS2/EPYC1 matrix condensates in the same lines depicted in **a**–**d**. Images are presented at the same scale. Arrowhead in **g** points to matrix-traversing membrane. **j**, SBF–SEM sections of a condensate in the *CrRBCS2;EPYC1-GFP;SAGA1-mCherry;MITH1-mCerulean* line and 3D reconstruction of the SBF–SEM data, highlighting matrix-traversing membranes in green. **k**, Example TEM image of the Arabidopsis line *CrRBCS2;EPYC1-GFP;MITH1-mCerulean* highlighting the appearance of two membrane sheets, each with its own lumen. Graph depicts grey values measured across a distance indicated by the blue line in the membrane zoom image.

**Extended Data Fig. 2:**
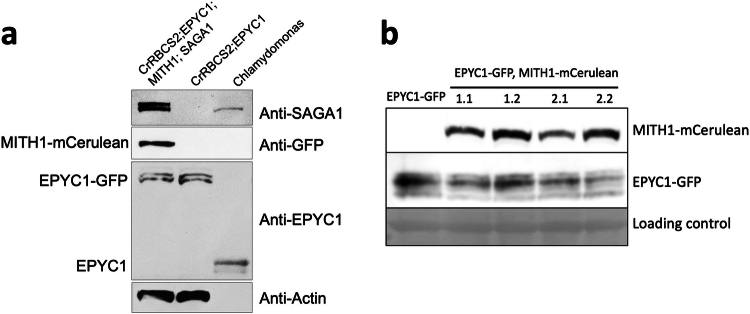
MITH1-mCerulean and SAGA1-mCherry were heterologously expressed in an Arabidopsis line containing CrRBCS1 and EPYC1-GFP. Western blots were performed on soluble protein extracts run through a denaturing SDS-PAGE gel to probe for a, proteins expressed in the Arabidopsis *CrRBCS2;EPYC1-GFP;MITH1-mCerulean;SAGA1-mCherry* line (lane 1) and *CrRBCS2;EPYC1-GFP* line (lane 2) as compared to the native proteins from Chlamydomonas (lane 3) and b, proteins expressed in the *CrRBCS2;EPYC1-GFP* line (lane 1) compared to *CrRBCS2;EPYC1-GFP;MITH1-mCerulean* plants (lanes 2-5). The anti-GFP antibody was used to detect mCerulean. In (**a**), Actin is used as a loading control for the Arabidopsis samples. In (**b**), a Ponceau stain is used to detect total protein as a loading control. Predicted molecular weights are as follows: SAGA1: 168 kDa, SAGA1-mCherry: 195 kDa, MITH1: 139 kDa, MITH1-mCerulean: 171 kDa, EPYC1: 32 kDa, EPYC1-GFP: 59 kDa.

**Extended Data Fig. 3:**
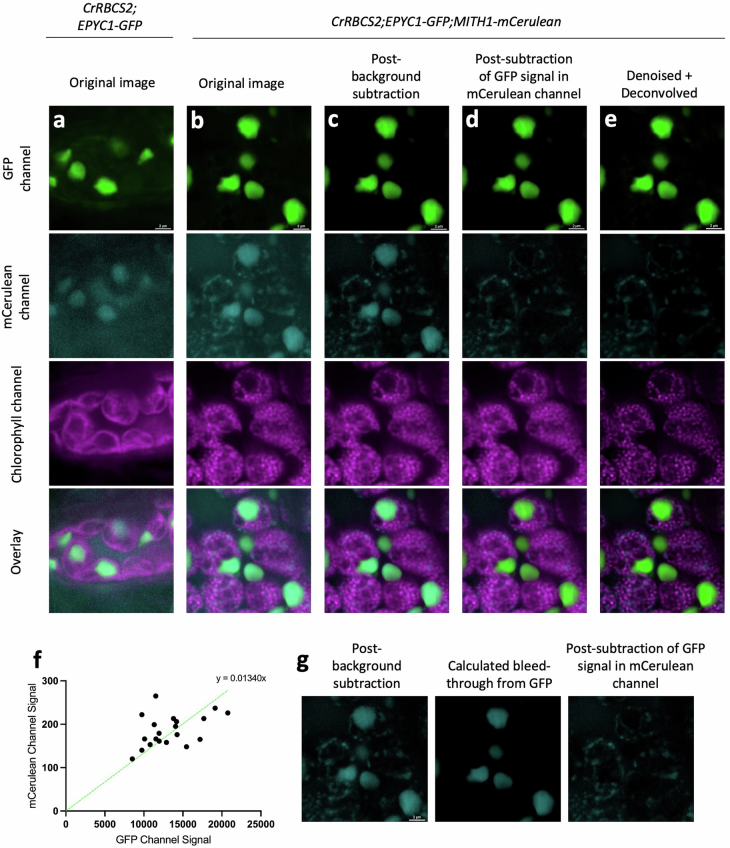
Calculated subtraction of bleedthrough from EPYC1-GFP to the MITH1-mCerulean channel. **a**, EPYC1-GFP bleeds into the mCerulean channel, as seen by the mCerulean signal present in the *CrRBCS2;EPYC1* line despite the absence of MITH1-mCerulean. **b**, Original images of the *CrRBCS2;EPYC1-GFP;MITH1-mCerulean* line. **c**, *CrRBCS2;EPYC1-GFP;MITH1-mCerulean* images following subtraction of a constant value for background signal in the GFP and mCerulean channels. **d**, *CrRBCS2;EPYC1-GFP;MITH1-mCerulean* images following subtraction of calculated bleedthrough of GFP signal into the mCerulean channel. **e**, *CrRBCS2;EPYC1-GFP;MITH1-mCerulean* images after further denoising and deconvolution as detailed in Methods. **f**, Scatter plot displaying the association between GFP signal intensity and mCerulean intensity in the *CrRBCS2;EPYC1-GFP* line (not expressing mCerulean); each dot represents the average mCerulean and GFP signals from a region in an EPYC1-GFP condensate. The slope of the regression was used to quantify the amount of bleedthrough from GFP into the mCerulean channel. **g**, Additional information on the calculated bleedthrough signal. From left to right, the mCerulean channel image from panel c is shown, followed by the calculated bleedthrough signal from the GFP channel into the mCerulean channel, followed by the image resulting from the subtraction of the calculated bleedthrough signal (this image corresponds to the image shown in the mCerulean channel of panel d).

**Extended Data Fig. 4:**
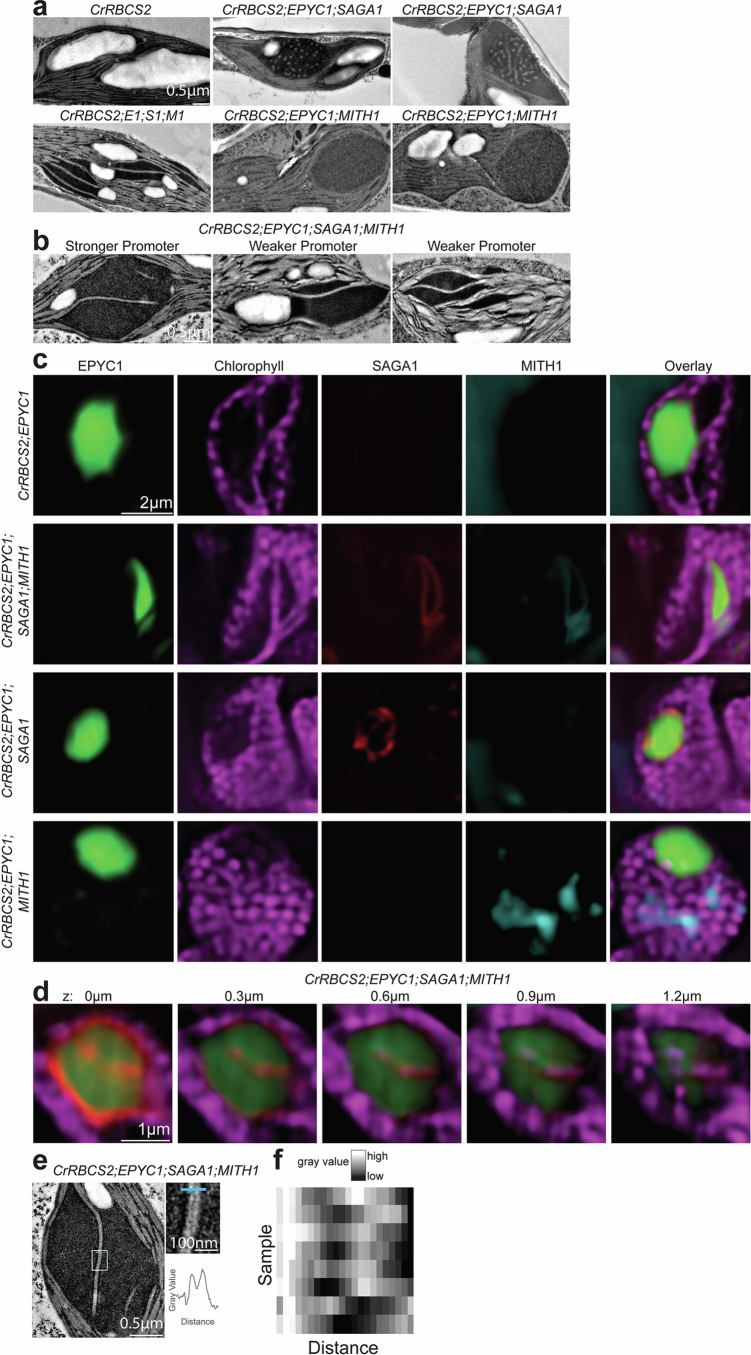
In Arabidopsis, SAGA1-mCherry and MITH1-mCerulean are both necessary to generate matrix-traversing membranes. **a**, TEM images of the chloroplasts of the CrRBCS2 line and lines expressing EPYC1-GFP, SAGA1-mCherry, and MITH1-mCerulean. **b**, Example images of the *CrRBCS2;EPYC1-GFP;SAGA1-mCherry;MITH1-mCerulean* line where MITH1 is expressed under either the stronger CaMV35S promoter or a weaker promoter, AtRbcS3B. **c**, Additional images of heterologous protein localizations in the Arabidopsis lines are shown. **d**, Z-stack depicting the overlay of channels for the *CrRBCS2;EPYC1-GFP;SAGA1-mCherry;MITH1-mCerulean* pyrenoid shown in Fig. 2e. **e**, Example TEM of the *CrRBCS2;EPYC1-GFP;SAGA1-mCherry;MITH1-mCerulean* highlighting the apparent presence of a double membrane. Gray values were measured across the blue line and were used to produce a graph plotting the gray value across the line. **f**, Heatmap depicting gray values measured for 8 traversing membranes in different cells. Within each panel, all images are shown at the same scale.

To determine whether MITH1 and SAGA1 are sufficient for producing pyrenoid matrix-traversing membranes, we expressed the two proteins in the heterologous system of *Arabidopsis thaliana* plants (Fig. 2 and Extended Data Figs. 2–4). While wild-type Arabidopsis chloroplasts lack a pyrenoid, a synthetic Chlamydomonas-like pyrenoid matrix was previously reconstituted in Arabidopsis chloroplasts by expressing the linker protein EPYC1-GFP and the EPYC1-binding Chlamydomonas Rubisco small subunit CrRBCS2 (Fig. 2a,f and Extended Data Fig. 4c)^9^. Here we transformed these *CrRBCS2;EPYC1-GFP* plants containing a synthetic matrix with *SAGA1-mCherry* and chloroplast-targeted *MITH1-mCerulean* (Fig. 2b–e,g–i and Extended Data Fig. 4). Whereas membranes had never previously been observed within condensates of the parental *CrRBCS2;EPYC1-GFP* Arabidopsis line^9^ (Fig. 2a), we detected chlorophyll fluorescence streaks inside the condensates of the *CrRBCS2;EPYC1-GFP;SAGA1-mCherry;MITH1-mCerulean* line, suggesting that they contain thylakoid membranes (Fig. 2b,e). TEM confirmed that membranes traverse the matrix condensates in the *CrRBCS2;EPYC1-GFP;SAGA1-mCherry;MITH1-mCerulean* line (Fig. 2g and Extended Data Fig. 4a,b). We observed traversing membranes in 32% of *CrRBCS2;EPYC1-GFP;SAGA1-mCherry;MITH1-mCerulean* condensates (*n* = 166; Fig. 2g), but in none in the condensates of the *CrRBCS2;EPYC1-GFP* line (*n* = 117; *P* < 0.0001, Fisher’s exact test; Fig. 2f). These results demonstrate that SAGA1 and MITH1 together are sufficient to generate matrix-traversing thylakoid membranes in a heterologous system.

Neither SAGA1 nor MITH1 alone was sufficient to produce traversing membranes when expressed in the *CrRBCS2;EPYC1-GFP* Arabidopsis line (Fig. 2c,d,h,i). In a separate manuscript focused on reconstituting a pyrenoid starch sheath in Arabidopsis^14^, extensive characterization of the *CrRBCS2;EPYC1-GFP;SAGA1-mCherry* line established that its condensates contain starch granules as well as a network of lower-electron-density regions that contain SAGA1 but not membranes (Fig. 2h). When we additionally expressed MITH1 in this line, we no longer observed this network or starch granules in the condensates (Fig. 2g). Since neither the network nor starch granules inside the matrix are present in wild-type Chlamydomonas cells, we favour the view that both features are aberrant consequences of SAGA1 expression in the absence of other relevant Chlamydomonas proteins.

In the *CrRBCS2;EPYC1-GFP;SAGA1-mCherry;MITH1-mCerulean* line, traversing membranes appeared as straight lines that typically spanned the entire length of the matrix in the TEM cross-sections (Fig. 2g and Extended Data Fig. 4a,b), suggesting that the matrix-traversing membranes in Arabidopsis are arranged in sheets. Three-dimensional (3D) reconstruction of serial block-face scanning electron microscopy data further demonstrated that the traversing membranes were arranged in sheets that in some instances spanned more than 750 nm in width and depth (Fig. 2j and Supplementary Videos 1–5). TEM suggested that the traversing membranes typically consisted of two parallel thylakoid-like sheets, each with its own lumen (Fig. 2k and Extended Data Fig. 4e,f).

While the sheet-like morphology we observed in Arabidopsis was distinct from the tubular morphology of the Chlamydomonas matrix-traversing membranes (Fig. 1b,c), it was remarkably similar to that of the most-commonly-observed morphology of matrix-traversing membranes in most other algal species, including those of hornworts^15^, chlorella^16^, diatoms^17^ and chlorarachniophytes^18^. Our observations therefore indicate that the process of causing membrane sheets to traverse a matrix is distinct from the process of shaping those membranes into tubules, and that while SAGA1 and MITH1 appear to not be sufficient to shape membranes into tubules, they are both necessary and sufficient to induce membrane sheets to traverse a matrix.

The discovery of proteins that are both necessary and sufficient for the formation of matrix-traversing membranes now enables investigation in Chlamydomonas and Arabidopsis of how these proteins produce matrix-traversing membranes.

## MITH1 and SAGA1 function at matrix-traversing membranes

**Fig. 3:**
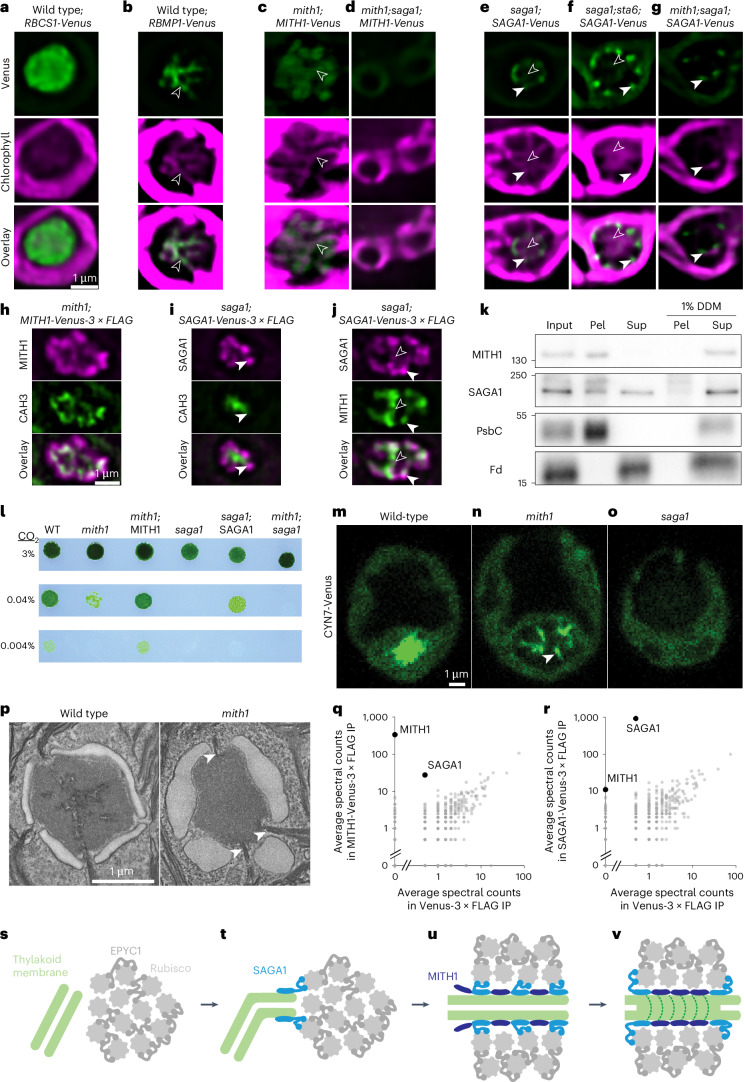
SAGA1 and MITH1 localize to membranes, bind each other and perform distinct functions in matrix-traversing membrane biogenesis. **a**–**g**, Localizations of Venus-tagged proteins in various Chlamydomonas mutant backgrounds. Transparent arrowheads point to membranes inside the matrix; filled arrowheads point to membranes at the matrix entry sites. Images are presented at the same scale. **h**–**j**, Two-colour immunofluorescence was performed for anti-FLAG and anti-CAH3 (**h**,**i**) and anti-FLAG and anti-MITH1 (**j**). Images are presented at the same scale. **k**, Cell fractionation and western blot in the absence and presence of 1% DDM detergent; Pel, pellet; Sup, supernatant; Fd, ferredoxin. Representative images from 3 biological repeats are shown. **l**, Spot test depicting growth at varying levels of CO_2_ and 100 µmol photons m^−2^ s^−1^ light levels. **m**–**o**, Localization of CYN7-Venus in wild-type and mutant backgrounds. Images are presented at the same scale. **p**, TEM of wild-type and *mith1* mutant pyrenoids. Arrowheads point to matrix surface-associated membranes. **q**,**r**, Spectral counts of proteins identified by mass spectrometry after immunoprecipitation of MITH1-Venus-3×FLAG (**q**) or SAGA1-Venus-3×FLAG (**r**), plotted against the spectral counts of the same proteins after immunoprecipitation of Venus-3×FLAG as a control for non-specific binding. Non-labelled points are partially transparent to facilitate visualization of overlapping points. Results from 2 replicates were averaged. **s**–**v**, Model for how SAGA1 and MITH1 produce matrix-traversing membranes. **s**, Without either protein, membranes are unable to enter the Rubisco matrix. **t**, SAGA1 initiates contact between thylakoid membrane sheets and the surface of the pyrenoid by binding to membranes and Rubisco. **u**, SAGA1 recruits MITH1, which increases the binding force per unit area between membranes and matrix, outcompeting EPYC1–Rubisco interactions and allowing extension of membrane sheets into the matrix. **v**, In Chlamydomonas, additional factors shape the membrane sheets into cylindrical tubules.

**Extended Data Fig. 5:**
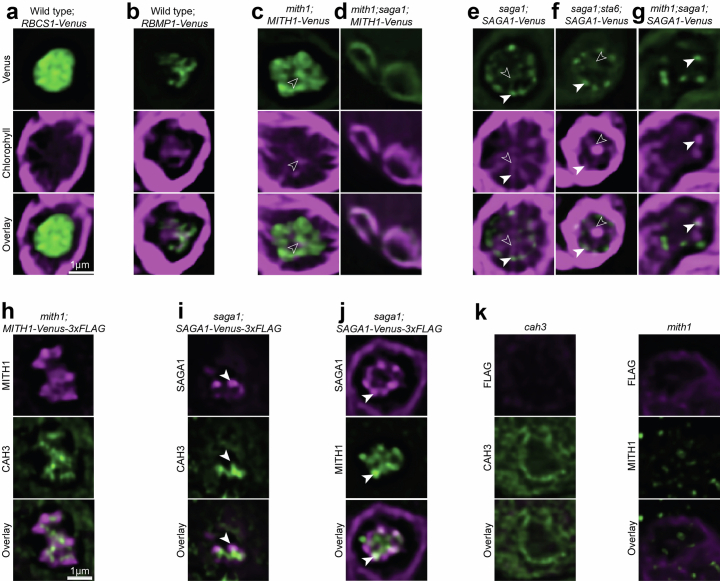
Additional images of SAGA1 and MITH1 localizing to the matrix-traversing membranes. **a-g**, Localizations of Venus-tagged proteins in various Chlamydomonas mutant backgrounds. Transparent arrowheads point to membranes inside the matrix; filled arrowheads point to membranes at the matrix entry sites. Images are presented at the same scale. **h-j**, Two-color immunofluorescence was performed for anti-FLAG and anti-CAH3 (h,i) and anti-FLAG and anti-MITH1 (**j**). Images are presented at the same scale. **k**, Immunofluorescence controls showing background signal in the region of the pyrenoid. Images are presented at the same scale as in (**h**). Control images are thresholded such that their intensity ceiling is matched to the experiment(s) which used that combination of antibodies. For the cah3 control, the FLAG and CAH3 channels were thresholded so that their upper intensity limit matched that of the FLAG and CAH3 images showing the weakest signal across Fig. 3h, i and Extended Data Fig. 5h, i. For the mith1 control, the FLAG and MITH1 channels were thresholded so that their upper intensity limit matched that of the FLAG and MITH1 images showing the weakest signal across Fig. 3j and Extended Data Fig. 5j.

We examined the localization of MITH1-Venus in Chlamydomonas using the complemented *mith1;MITH1-Venus* background, where the tagged protein is functional (Fig. 1e). Whereas MITH1 was previously proposed to localize to the Chlamydomonas pyrenoid matrix^19,20^, here we observed that it localizes to and functions at the matrix-traversing membranes. MITH1 localized in a branching pattern in the pyrenoid similar to that of RBMP1, a known matrix-traversing membrane protein^21^, and distinct from the homogeneous localization of the Rubisco small subunit (RBCS1), which is known to occupy the matrix^7^ (Fig. 3a–c and Extended Data Fig. 5a–c). MITH1 co-localized with faint chlorophyll fluorescence from the matrix-traversing membranes (Fig. 3c and Extended Data Fig. 5c) and with CAH3, a matrix-traversing membrane lumen-localized carbonic anhydrase^22^ (Fig. 3h and Extended Data Fig. 5h). We observed MITH1 fluorescence signal in a broader region than RBMP1, chlorophyll or CAH3 (Fig. 3b,c,h and Extended Data Fig. 5b,c,h). This observation could be due to MITH1 taking up space on the outside of the membranes, whereas RBMP1 is a predicted transmembrane protein, chlorophyll is probably associated with transmembrane proteins, and CAH3 is in the lumen. Chlamydomonas cell fractionation also supported membrane localization, as MITH1 co-pelleted with membranes in the absence of detergent and was solubilized when detergent was added (Fig. 3k). MITH1 localized to the matrix-traversing membranes in *CrRBCS2;EPYC1-GFP;SAGA1-mCherry;MITH1-mCerulean* Arabidopsis (Fig. 2e and Extended Data Fig. 4c), further supporting a membrane localization. Together, these results demonstrate that MITH1 localizes to the matrix-traversing membranes when SAGA1 is present.

We had previously found that SAGA1-Venus localizes to puncta and streaks at the Chlamydomonas pyrenoid periphery, and had hypothesized that this localization was mediated by its CBM20 starch-binding domain^13^, which could bind the starch sheath that surrounds the Chlamydomonas pyrenoid (Fig. 1a). However, here we observed that SAGA1 localized to pyrenoid-periphery puncta even in the starchless *sta6* mutant background (Fig. 3e,f and Extended Data Fig. 5e,f), indicating that SAGA1 does not require starch for its normal localization.

We observed that the Chlamydomonas SAGA1-Venus puncta co-localized with the outer extremities of the matrix-traversing membranes as visualized through chlorophyll streaks (Fig. 3e,f and Extended Data Fig. 5e,f), CAH3 (Fig. 3i and Extended Data Fig. 5i) and MITH1 (Fig. 3j and Extended Data Fig. 5j). In the *saga1;SAGA1-Venus* strain, we observed a gap between the SAGA1-Venus fluorescence and the chlorophyll fluorescence outside the pyrenoid, which we did not observe in the starchless *saga1;sta6;SAGA1-Venus* strain (Fig. 3e,f and Extended Data Fig. 5e,f), indicating that the prominent SAGA1 puncta and streaks were at the sites where the matrix-traversing membranes enter the pyrenoid matrix on the inner face of the starch granules. Furthermore, we observed faint SAGA1 signal deeper inside the pyrenoid, in matrix-traversing streaks that co-localized with the matrix-traversing chlorophyll streaks (Fig. 3f and Extended Data Fig. 5f, transparent arrowheads) and with MITH1 (Fig. 3j, transparent arrowheads). In the cell fractionation experiment, SAGA1 was present both in the membrane pellet and the supernatant, and the proportion in the supernatant increased upon addition of detergent, suggesting that while SAGA1 interacts with membranes, a portion of the protein dissociates from membranes during the experiment or exists in a distinct soluble state (Fig. 3k). Our results indicate that in Chlamydomonas, SAGA1 localizes throughout the matrix-traversing membranes and is enriched in puncta at their periphery.

Further supporting a matrix-traversing membrane localization of SAGA1, in the *CrRBCS2;EPYC1-GFP;SAGA1-mCherry;MITH1-mCerulean* Arabidopsis line, SAGA1-mCherry localized along the EPYC1-GFP condensate-traversing chlorophyll streaks (Fig. 2e and Extended Data Fig. 4c). A notable difference from Chlamydomonas was that in the Arabidopsis system, SAGA1 signal was homogeneous across the matrix-traversing membranes, while in Chlamydomonas SAGA1 was enriched in puncta at the periphery of the pyrenoid and only faint SAGA1 signal was observed in the matrix-traversing membranes deeper inside the pyrenoid (Fig. 3e,i,j). One possible explanation for this observation is that SAGA1 may have a preference for localization to sheet-like membranes over tubules. Thus, in Chlamydomonas, SAGA1 would preferentially localize to the peripheral regions of the matrix-traversing membranes, where these membranes transition from sheets into tubules (Fig. 1b), but in Arabidopsis, it would localize throughout the matrix-traversing membranes, as they are sheets along their full length (Fig. 2j). In contrast to SAGA1, MITH1 localized throughout the matrix-traversing membranes in both Arabidopsis (Fig. 2e) and Chlamydomonas (Fig. 3c,h), which could potentially reflect a different membrane curvature preference from that of SAGA1. We conclude that MITH1 and SAGA1 localize to and function at the matrix-traversing membranes.

## MITH1 requires SAGA1 for localization and function

**Extended Data Fig. 6:**
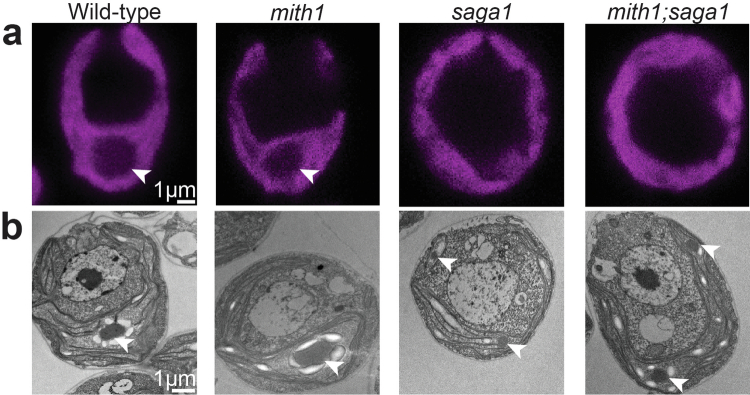
Comparisons of cell morphology between wild-type, *mith1*, *saga1*, and *mith1;saga1* mutants. Morphology was assayed using **a**, microscopy of chlorophyll autofluorescence or **b**, TEM. Arrowheads point to observed pyrenoids. All images in each row are shown at the same scale.

The Chlamydomonas *saga1* mutant displayed a more-severe growth defect under air CO_2_ conditions than the *mith1* mutant (Fig. 3l). The difference in the severity of the mutant growth defects suggests that the two proteins play distinct roles in producing matrix-traversing membranes. To determine the order in which SAGA1 and MITH1 act to produce matrix-traversing membranes, we tested their epistasis by generating a *mith1;saga1* double mutant. We compared the growth and cell morphology of the single and double mutants using spot tests, light microscopy and TEM. The *mith1*;*saga1* double mutant displayed similar growth and morphological defects to the *saga1* single mutant (Fig. 3l and Extended Data Fig. 6), indicating that *saga1* is epistatic to *mith1* and suggesting that MITH1 requires SAGA1 to function.

**Extended Data Fig. 7:**
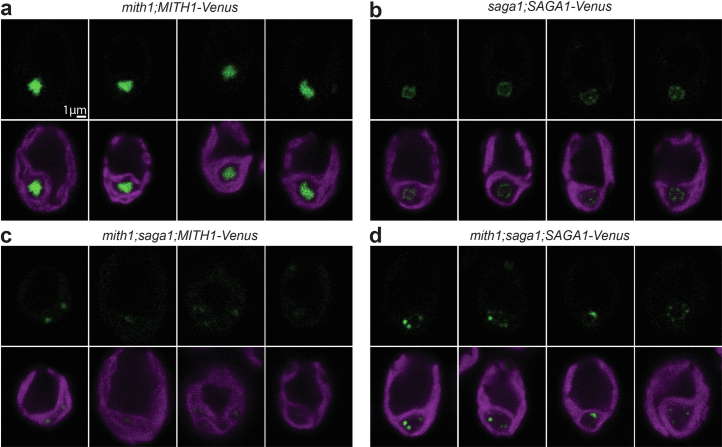
MITH1 localization is dependent on SAGA1, but SAGA1 localizes independently of MITH1. **a**, MITH1 in the mith1;MITH1-Venus complemented strain localizes to structures within the pyrenoid. **b**, SAGA1 in the *saga1;SAGA1-Venus* complemented strain localizes to puncta at the pyrenoid periphery16. **c**, MITH1 mis-localizes throughout the chloroplast in the absence of SAGA1. **d**, SAGA1 localizes to pyrenoid-peripheral puncta in the absence of MITH1. All images in this figure are presented at the same scale.

Consistent with this dependence, we found that when we expressed each Venus-tagged protein in a *mith1;saga1* double mutant, SAGA1 still localized to the pyrenoid in the absence of MITH1, but not vice-versa (Fig. 3d,g and Extended Data Fig. 7). When SAGA1-Venus was expressed without MITH1 in this background, it showed normal localization to pyrenoid puncta (Fig. 3g and Extended Data Fig. 7). In contrast, in the absence of SAGA1, MITH1-Venus mislocalized throughout the chloroplast (Fig. 3d and Extended Data Fig. 7).

Consistent with observations in Chlamydomonas, in Arabidopsis plants that lacked MITH1-mCerulean, SAGA1-mCherry still localized to the EPYC1-GFP condensate (Fig. 2c and Extended Data Fig. 4c), but in plants that lacked SAGA1, MITH1-mCerulean was dispersed throughout the chloroplast (Fig. 2d and Extended Data Fig. 4c). Considering the absence of other pyrenoid factors in the Arabidopsis system, these results show that SAGA1 is not only necessary but also sufficient for recruiting MITH1 to the EPYC1-Rubisco condensate. Altogether, our findings demonstrate that whereas SAGA1 can localize to the EPYC1-Rubisco condensate independently of MITH1, MITH1 requires SAGA1 for its recruitment to the condensate and for its function.

## SAGA1 is necessary for initiation of traversing membranes

**Extended Data Fig. 8:**
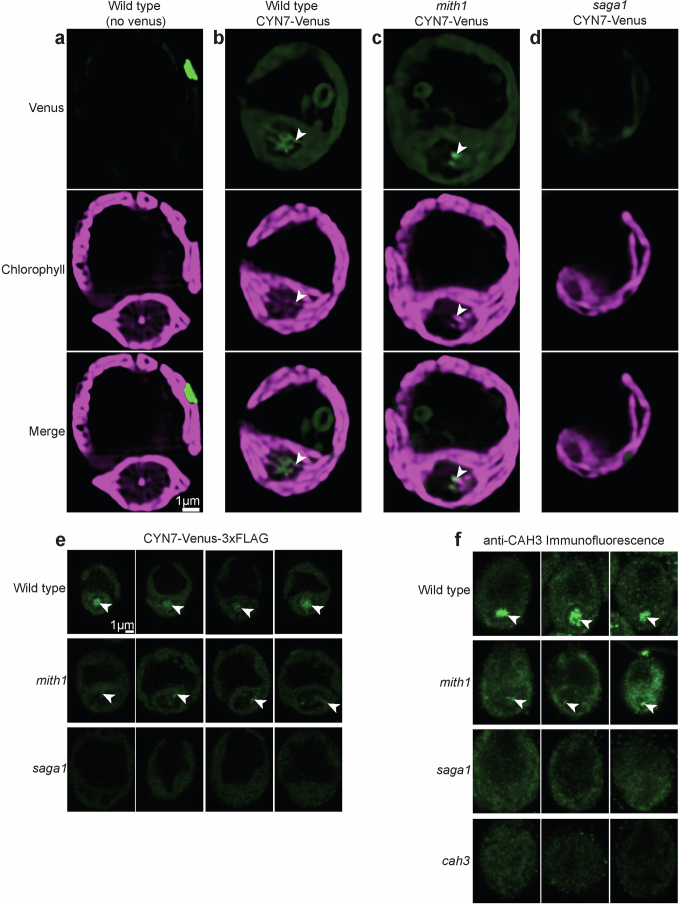
Matrix-traversing membrane-localized proteins CYN7 and CAH3 are mis-localized in mith1 and saga1 mutants. **a-d**, Images of Venus and chlorophyll channels in a wild-type control not expressing Venus (**a**), a wild-type strain expressing CYN7-Venus (**b**), *mith1* expressing CYN7-Venus, and *saga1* expressing CYN7-Venus (**d**). Images are presented at the same scale. Arrowheads point to the wild-type matrix-traversing membrane tubules and *mith1* pyrenoid-associated membranes labeled by CYN7-Venus. **e**, Additional images of CYN7-Venus expression in live cells of the wild-type, *mith1*, and *saga1* strains. Arrowheads point to bright CYN7-Venus signal associated with the pyrenoid. **f**, Immunofluorescence with an anti-CAH3 antibody was used to visualize localization of endogenous CAH3. Arrowheads point to bright CAH3 signal associated with the pyrenoid. In *mith1* mutants, CAH3 localized to small puncta and streaks at the periphery of the pyrenoid rather than to the star-like traversing network seen in wild-type cells. The *cah3* mutant was used as a negative control. Images are presented at the same scale as (**e**).

To distinguish the roles of SAGA1 and MITH1 in matrix-traversing membrane biogenesis, we characterized matrix-traversing membrane defects in the Chlamydomonas *saga1* and *mith1* mutants using the protein CYN7-Venus as a marker for matrix-traversing membranes (Fig. 3m–o). CYN7 (Cre12.g544150) is a predicted thylakoid luminal peptidyl-prolyl isomerase that was shown to be enriched in the matrix-traversing membranes of wild-type cells^23^, a localization that we confirmed here (Fig. 3m and Extended Data Fig. 8a,b). In the *saga1* mutant, CYN7-Venus was dispersed throughout the chloroplast (Fig. 3o and Extended Data Fig. 8d,e), demonstrating that a normal matrix-traversing membrane network was not present in cells of this mutant and indicating that SAGA1 is necessary for initiation of matrix-traversing membranes.

## MITH1 is necessary for extension of traversing membranes

Unlike in the *saga1* mutant, in the *mith1* mutant, CYN7-Venus localized to streaks and puncta associated with the periphery of the pyrenoid (Fig. 3n and Extended Data Fig. 8c,e). These observations suggest that pyrenoid membrane formation initiates at the correct cellular location in the *mith1* mutant but then stalls before these membranes are extended through the pyrenoid matrix. Consistent with this idea, several TEM images of the *mith1* mutant showed thylakoid membranes crossing the pyrenoid starch sheath and stopping at the surface of the matrix (Fig. 3p, arrowheads). SAGA1-Venus’s punctate localization when expressed in the double mutant (Fig. 3g) suggests that SAGA1 is present in these streaks and functions to form them.

## SAGA1 and MITH1 physically interact

The results presented so far indicate that SAGA1 and MITH1 together are necessary and sufficient to cause membranes to traverse the matrix, and that both proteins localize to these membranes. To understand how SAGA1 and MITH1 cause membranes to traverse the matrix, we sought to understand the proteins’ interactions with matrix and with each other.

SAGA1 physically interacts with both Rubisco large and small subunits by yeast two-hybrid assay^13^, and it contains two Rubisco-binding motifs whose binding to Rubisco we previously confirmed in vitro^21^. We could not detect evidence of direct MITH1 physical interaction with Rubisco or EPYC1 by yeast two-hybrid assay, and the lack of an interaction is supported by the absence of detectable MITH1 in the EPYC1-Rubisco condensates of the *CrRBCS2;EPYC1;MITH1* lines (Fig. 2d and Extended Data Fig. 4c).

To test whether SAGA1 can bind MITH1 in Chlamydomonas, we performed immunoprecipitation–mass spectrometry with SAGA1 and MITH1 as bait proteins. SAGA1 was MITH1’s most-abundant specific interactor, and MITH1 was one of SAGA1’s most-specific interactors, indicating that the two proteins physically interact (Fig. 3q,r and Supplementary Table 5). Taken together with our observation that MITH1 requires SAGA1 for localization to the pyrenoid in the Arabidopsis system (Fig. 2b,d), these results suggest that SAGA1 recruits MITH1 to the pyrenoid via direct binding.

## A proposed model for matrix-traversing membrane biogenesis

On the basis of the above results, we propose a model for how SAGA1 and MITH1 cause thylakoid membranes to traverse the pyrenoid matrix (Fig. 3s–v). We posit that in the absence of SAGA1 and MITH1, it is energetically unfavourable for thylakoid membranes to traverse the matrix, as this would disrupt Rubisco-EPYC1 bonds in the matrix (Fig. 3s). To allow a membrane to traverse through the matrix, Rubisco-EPYC1 bonds that would otherwise be in the path of the traversing membrane must be replaced with favourable Rubisco-membrane bonds. We propose that SAGA1 and MITH1 together provide such Rubisco-membrane bonds, making it energetically favourable for membranes to traverse the matrix (Fig. 3u).

SAGA1 is necessary for the initiation of matrix-traversing membranes as recognized by CYN7 (Fig. 3m–o) and CAH3 (Extended Data Fig. 8). We propose that SAGA1 mediates an initial contact between thylakoids and matrix (Fig. 3t) by binding to Rubisco^13,21^ and membranes (Figs. 2e and 3e,i,k). In the absence of MITH1, SAGA1 does not appear to produce a sufficient membrane–matrix adhesive force per unit area to displace Rubisco-EPYC1 bonds, and thylakoid membranes contact the matrix but do not penetrate into it (Fig. 3g,n,p and Extended Data Fig. 8). SAGA1 allows membranes in contact with the matrix to acquire matrix-traversing membrane identity as recognized by CYN7 (Fig. 3m–o). We propose that MITH1 increases the membrane–matrix adhesive force per unit area by binding to SAGA1 (Fig. 3q,r) and membranes (Figs. 2e and 3c,h,k), making it energetically favourable for membranes to traverse the matrix. In the absence of SAGA1, MITH1 cannot contribute to membrane–matrix interactions, as it lacks any detectable Rubisco-binding motif^21^ and appears to be unable to directly bind Rubisco or EPYC1 (Fig. 2d and Extended Data Fig. 4c). The molecular, structural and biophysical aspects of this proposed mechanism are an exciting area for continued research.

The observation that expression of SAGA1 and MITH1 in Arabidopsis produced sheets (Fig. 2j) rather than tubules suggests that in Chlamydomonas, additional factors, which remain to be discovered, act separately from SAGA1 and MITH1 to promote membrane curving into tubules (Fig. 3v). The search for these factors and their eventual characterization is another exciting area for future work.

While matrix-traversing membrane biogenesis is independent of starch sheath biogenesis as evidenced by the presence of matrix-traversing membranes in starchless mutants^24^, SAGA1’s starch-independent enrichment at the extremities of the matrix-traversing membranes (Fig. 3e,f) places its CBM20 starch-binding domain in close proximity to the starch sheath, whose biogenesis it could help regulate. Indeed, expressing SAGA1 in Arabidopsis expressing CrRBCS and EPYC1 led to increased occurrence of starch in proximity to the Rubisco-EPYC1 matrix^14^. Starch-binding protein SAGA2 (Cre09.g394621)^21^ shares weak sequence similarity with SAGA1 and MITH1 (ref. ^11^), but it is unlikely to contribute to matrix-traversing membrane biogenesis based on the growth phenotypes of the *saga2* mutant^11^, localization of SAGA2 away from membranes^21^ and the absence of matrix-traversing membranes in Arabidopsis plants expressing SAGA2 in addition to SAGA1, EPYC1 and CrRBCS^14^.

## Matrix-traversing membrane-deficient mutants have multiple matrix condensates

**Fig. 4:**
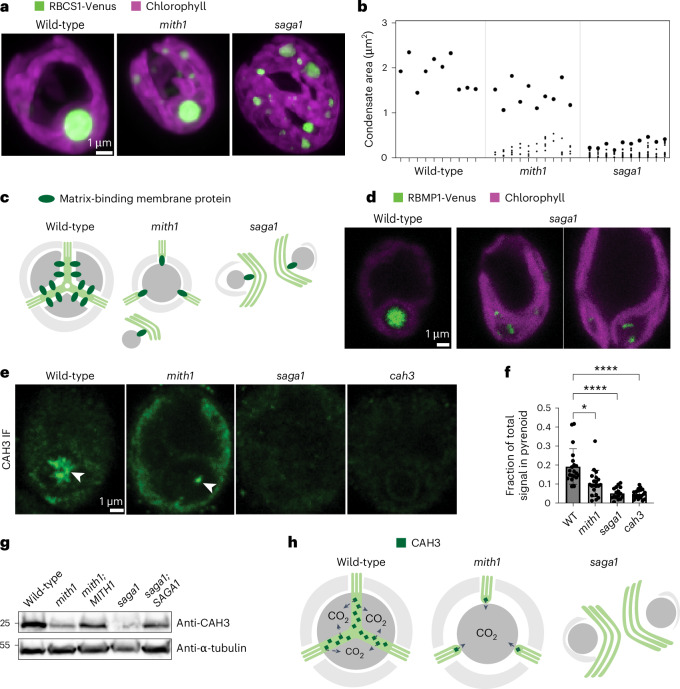
Matrix-traversing membrane-deficient mutants have multiple pyrenoids and defective CO_2_ delivery to the pyrenoid. **a**, Max-*z* projections depicting RBCS1-Venus localization in Chlamydomonas wild-type, *mith1* and *saga1* cells. The chloroplast is visualized by chlorophyll autofluorescence. **b**, Graph depicting the largest cross-sectional area observed in any *z*-slice for each condensate in each cell. Each tick on the *x* axis corresponds to a single cell; each point on the graph plots the area for a single condensate. The point depicting the largest condensate in each cell is enlarged to aid visualization. Data for 10 cells per strain are shown. **c**, Model: in the absence of normal matrix-traversing membranes, their resident matrix-binding proteins could mislocalize and nucleate Rubisco puncta elsewhere in the chloroplast. **d**, Localization of matrix-binding membrane protein RBMP1 in wild-type versus *saga1* mutant backgrounds. **e**, Anti-CAH3 immunofluorescence. Arrowheads point to regions of strong CAH3 signal associated with the pyrenoid. **f**, Quantification of fluorescence signal in anti-CAH3 immunofluorescence samples. Results depict the fraction of a cell’s total fluorescence signal found in the pyrenoid. Kruskal–Wallis followed by Dunn’s multiple comparisons test were used for statistical analyses. *n* = 20 cells per strain except for *saga1* with *n* = 16 cells. *P < 0.05, ****P < 0.0001. See Methods for exact *P* values. Mean ± s.d. **g**, Anti-CAH3 western blot performed on whole cell lysates. Anti-alpha-tubulin was used as a loading control. Representative results of 3 biological repeats are shown. **h**, Model: partially localized CAH3 provides enough CO_2_ to sustain partial growth in the *mith1* mutant.

Our discovery of mutants deficient in matrix-traversing membranes provides an opportunity to examine the impacts that these membranes have on other aspects of pyrenoid biogenesis. We had previously observed that the *saga1* mutant formed an average of 10 matrix condensates throughout the chloroplast (Fig. 4a) and had proposed that this was a consequence of starch defects^13^. Our discovery that SAGA1 mediates matrix-traversing membrane biogenesis raised the alternative hypothesis that the multiple matrix condensates in the *saga1* mutant are, instead, a consequence of defects in matrix-traversing membranes.

**Extended Data Fig. 9:**
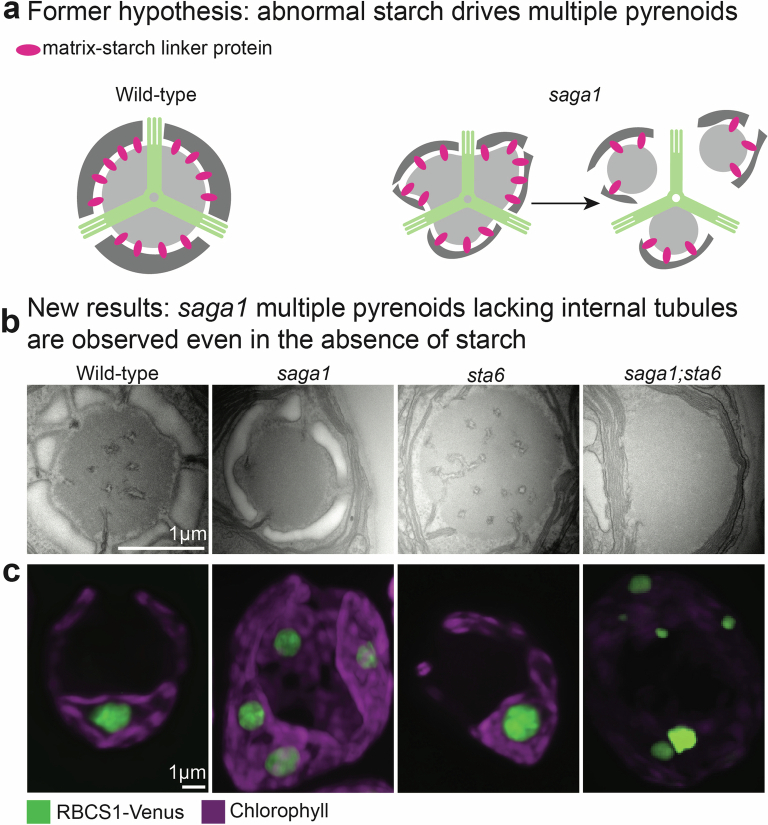
*saga1* mutants have multiple pyrenoids and lack matrix-traversing membranes even in the absence of starch. **a**, In an earlier model^13^, we hypothesized that an increased surface area of misshapen starch leads to multiple pyrenoids which are missing matrix-traversing membranes in the saga1 mutant. In that model, abnormal starch causes the formation of multiple matrix condensates; matrix-starch linker proteins mediate the influence of starch on matrix morphology and condensate number. **b**, TEM images of wild-type and mutant pyrenoids, including the starchless *sta6* mutant and the *saga1;sta6* double mutant. Previous work^24,47^ found that the starchless *sta6* mutant has a functional pyrenoid in growth conditions similar to the ones used in this study. **c**, Fluorescence images of RBCS1-Venus expressed in the wild type compared to mutant backgrounds. All images in each row are shown at the same scale.

To test our original hypothesis that the multiple matrix condensates in the *saga1* mutant are a consequence of abnormal starch, we generated and examined a starchless *saga1;sta6* double mutant. The *saga1;sta6* double mutant still exhibited multiple matrix condensates (Extended Data Fig. 9), demonstrating that the multiple matrix condensates in the *saga1* mutant are not caused by starch defects and favouring the alternative hypothesis that the multiple condensates are a consequence of abnormal matrix-traversing membranes in this mutant.

Further consistent with this alternative hypothesis, we also observed multiple matrix condensates in the *mith1* mutant (Fig. 4a), which has an apparently normal starch sheath but has clear defects in matrix-traversing membranes. The *mith1* multiple-condensates phenotype was less pronounced than that of *saga1*, with a single major pyrenoid present and fewer aberrant condensates (Fig. 4b). Considering that *mith1* mutants also have less-pronounced defects in matrix-traversing membrane biogenesis, as they initiate matrix-traversing membranes while *saga1* mutants do not (Fig. 3m–o), our observations indicate that the extent of the multiple-matrix-condensates phenotype is correlated with the extent of matrix-traversing membrane biogenesis defects. Taken together, these data support the hypothesis that matrix-traversing membrane defects lead to multiple matrix condensates.

One potential explanation for how the extra condensates are formed in the *saga1* and *mith1* mutants is that these condensates are nucleated by mislocalized tubule-resident proteins such as RBMP1/BST4, which is neither necessary nor sufficient for producing matrix-traversing membranes^25^ but has the ability to bind Rubisco^21^ (Fig. 4c). Consistent with this hypothesis, in the *saga1* mutant, RBMP1 mislocalized to multiple streaks at the periphery of chlorophyll voids throughout the chloroplast, which probably correspond to the pyrenoid matrix condensates (Fig. 4d and Extended Data Fig. 9c). Our results suggest a previously unappreciated role for pyrenoid membranes in establishing a single pyrenoid condensate.

## Matrix-traversing membranes enable growth under limiting CO_2_

Matrix-traversing membranes have long been proposed to deliver concentrated CO_2_ to the pyrenoid^26,27^, but their functional importance in CO_2_ delivery had not been experimentally established due to the lack of mutants known to specifically affect them. Our discovery that *mith1* and *saga1* mutants are deficient in matrix-traversing membrane biogenesis now allows us to examine the consequences of matrix-traversing membrane defects on pyrenoid function. These mutants exhibit growth defects in CO_2_-limiting conditions that are rescued by elevated CO_2_ (Fig. 3l)^11,13^, indicating that Rubisco in their pyrenoids is not supplied with sufficient CO_2_ to support growth in CO_2_-limiting conditions. Together, these observations provide experimental support for the proposed role of matrix-traversing membranes in delivering concentrated CO_2_ to the pyrenoid.

A potential explanation for why *mith1*’s growth defect is less severe than *saga1*’s at air levels of CO_2_ is that the partially formed pyrenoid membranes found at the surface of *mith1* mutant pyrenoids (Fig. 3n,p) may supply enough CO_2_ to sustain growth in air. In wild-type cells, the concentrated CO_2_ released by the matrix-traversing membranes is thought to be produced from bicarbonate (HCO_3_^−^) by the carbonic anhydrase CAH3 in the lumen of matrix-traversing membranes^26,27^. Therefore, if the partially formed pyrenoid membranes seen in *mith1* mutant pyrenoids have any capacity to supply CO_2_, we would expect CAH3 to be found there. Indeed, we observed that CAH3 localized to pyrenoid-peripheral puncta in the *mith1* mutants (Fig. 4e and Extended Data Fig. 8f), which probably correspond to the membrane protrusions seen by TEM (Fig. 3p). By contrast, in the *saga1* mutant, we could not detect CAH3 above background levels anywhere in the cell, indicating that *saga1* mutant pyrenoids lack a source of CO_2_ (Fig. 4e,f).

We found that CAH3 protein levels are decreased in both mutants, but to a more-severe extent in *saga1* (Fig. 4g). Furthermore, a recent study^28^ found that expression levels of other genes involved in carbon uptake, including HLA3 and LCIA, are decreased in the *saga1* mutant. Taken together, these observations indicate that matrix-traversing membranes are required for accumulation of proteins involved in CO_2_ and HCO_3_^−^ transport. We propose that this regulatory requirement avoids the unproductive and energetically wasteful operation of the CO_2_-concentrating mechanism when matrix-traversing membranes are not properly assembled and thus there is no effective route for delivery of CO_2_ to Rubisco. Low accumulation of CAH3 and other proteins needed for CO_2_ and HCO_3_^−^ transport could contribute to the growth defects seen in the *mith1* and *saga1* mutants (Figs. 3l and 4h). Our observations underscore the importance of matrix-traversing membranes for CO_2_ delivery and reveal the existence of a regulatory mechanism that ensures that concentrated CO_2_ is only released by properly assembled matrix-traversing membranes.

## Perspective

Our findings elucidate the biogenesis and functions of matrix-traversing membranes in the pyrenoid, an organelle that enhances aquatic photosynthetic CO_2_ assimilation. We have identified presumably the first biogenesis factors for matrix-traversing membranes, characterized how they function and demonstrated the necessity of matrix-traversing membranes for CO_2_ delivery. While the protein components may differ in other phylogenetic groups due to the recent convergent evolution of pyrenoids^2,29^, our work lays a blueprint for characterization of matrix-traversing membrane biogenesis across the phylogenetic tree and reveals basic principles that we propose apply broadly.

Matrix-traversing membranes have been hypothesized to localize the pyrenoid to the base of the chloroplast cup, as the matrix-traversing membrane tubules still form in the canonical location even in the absence of matrix or starch^7,30^. Our observation that *saga1* mutants fail to localize any of the pyrenoid’s three subcompartments to the base of the chloroplast further supports a role for matrix-traversing membranes in pyrenoid localization. The presence of a major pyrenoid at the canonical location in *mith1* mutants, which initiate pyrenoid membranes but fail to extend them into the matrix, indicates that the central portion of the matrix-traversing membranes is not needed for pyrenoid localization and suggests that the outer regions of the matrix-traversing membranes are critical for pyrenoid positioning.

Engineering a pyrenoid into crops has the potential to increase yields; however, efforts so far had been stalled by the inability to engineer matrix-traversing membranes^31–34^. Our discovery of matrix-traversing membrane biogenesis factors allowed us to overcome this major roadblock to pyrenoid engineering. By producing matrix-traversing thylakoid membranes in Arabidopsis, we achieved the reconstitution of all key structural features that we expect will be needed for a functional pyrenoid in a plant^31^. To achieve enhancement of CO_2_ fixation by synthetic pyrenoids in plants, over the coming decade, bicarbonate transporters and carbonic anhydrases will need to be engineered to deliver CO_2_ via the matrix-traversing membranes^31^. The plants we produced here are a powerful platform for testing additional pyrenoid components and advancing efforts to install CO_2_ delivery pathways.

In conclusion, the identification and molecular characterization of the factors that produce pyrenoid matrix-traversing membranes in Chlamydomonas not only advances the basic understanding of pyrenoid biogenesis, but more broadly, establishes a model for investigating the biophysics of how membranes are induced to traverse phase-separated condensates, enables the engineering of matrix-traversing membranes toward enhanced crop yields and brings us closer to a molecular understanding of the biological mechanisms that underlie the global carbon cycle.

## Methods

### Chlamydomonas strains and culture conditions

Strains used in this study are listed in Supplementary Table 1. Cells were maintained and cultured for experiments as described previously^21^. Briefly, unless otherwise noted, cells used in imaging and immunoblot experiments were grown to mid-log phase in Tris-phosphate (TP) liquid media in an orbital shaking incubator at room temperature (22 °C), 130 r.p.m., continuous cool white LED light at ∼175 µmol photons m^−2^ s^−1^ and in air enriched to 3% CO_2_ to enable growth of mutants with defective CO_2_ concentrating mechanisms. Approximately 16–24 h before experiments, cultures were moved to a different incubator with the same parameters except at air levels of CO_2_ to induce the CO_2_-concentrating mechanism. Cultures were collected for experiments at a cell density of ~2 × 10^6^ cells per ml, as measured by a Countess II Automated Cell Counter. The *mith1* mutant was PCR verified using an exon primer pair (TAGCCTGGACCGTGCTTAGT; ACCTTCATGTCCTTTGTGGC) that produces a 1 kb band from wild-type template DNA, and an insert primer pair (ACCTTCATGTCCTTTGTGGC; CTGCTGAGGCTGAATCTACTC) that produces a band from mutant template DNA.

### Crossing of Chlamydomonas strains

Several strains used in this study were produced through crossing opposite mating-type strains^35^. Cultures that were 5–6 days old and maintained on Tris-acetate-phosphate (TAP)-agar plates were transferred to N10 TAP plates, which are nitrogen-depleted, containing 10% of the nitrogen normally supplied on TAP plates. After 3 days on N10 plates, cells were transferred to a 50 ml Erlenmeyer flask containing 2.5 ml sterile distilled water using a sterile loop and agitated for 2 h on an orbital shaker in air at ∼175 µmol photons m^−2^ s^−1^ of light. Equal volumes of the mating type ‘+’ and mating type ‘–’ strains were mixed and left at ∼175 µmol photons m^−2^ s^−1^ of light without agitation for 0.5–1 h. Of the mixed cells, 300 µl were then plated on TAP-3% agar plates and air-dried in a sterile hood. The plates were then wrapped in foil and left in the dark for 5–7 days. After this time, vegetative cells were scraped from the plate with a flame-sterilized dull scalpel, leaving zygospores behind. Zygospores were allowed to mature into colonies and then transferred and streaked to single colonies on a new plate containing antibiotics to select for the correct genotype. For crosses to RBCS1-Venus or the starchless *sta6* mutant, further screening was used to search for fluorescence (using Typhoon scanner) or the absence of starch (tested with Lugol’s iodine treatment). The genotype was verified by PCR.

### Arabidopsis plant material and growth conditions

Arabidopsis (*Arabidopsis thaliana*, Col-0 background) strains used in this study are shown in Supplementary Table 2. Seeds were sown on compost or on half-strength Murashige and Skoog basal salt medium (Sigma) with 1% sucrose, stratified for 3 days at 4 °C and grown at 20 °C, ambient CO_2_ and 70% relative humidity under 200 µmol photons m^−2^ s^−1^ of light supplied by cool white LED lights (Percival SE-41AR3cLED, CLF PlantClimatics) in 12 h light/dark conditions or in a temperature-controlled greenhouse at 21 °C.

### Construct design and transformation

Plasmids used for Chlamydomonas are listed in Supplementary Table 3. Open reading frames were cloned into either the pLM005 (ref. ^3^) (Genbank: KX077945.1) or pRAM118 (ref. ^36^) (Genbank: MK357711) plasmid backbones. The two plasmid backbones are identical except that pLM005 contains an AphIII cassette encoding paromomycin resistance, which is replaced by the AphII cassette encoding hygromycin resistance in pRAM118. Both plasmids encode the fluorescent protein Venus followed by three copies of the FLAG epitope.

The MITH1-Venus-3×FLAG construct was made following a previously published PCR amplification protocol^20^. A KOD-Xtreme polymerase (Takara Bio) and manufacturer instructions were used to amplify *MITH1* from Chlamydomonas genomic DNA. MITH1 was annealed into an HpaI-digested pRAM118 vector using the InFusion kit (Takara Bio). PCR primers (ACAACAAGCCCAGTTATGTTGCGCTCTGCAAAGCC; ACCCAGATCTCCGTTGGGCGTCTTGAGAACGCTGTTGC) containing homology tails to pRAM118 were designed according to a previous protocol^20^. The plasmid’s sequence was verified by nanopore sequencing (CD Genomics) and was found to contain 6 point mutations in intron sequences in comparison to the reference Chlamydomonas v.5.6 genome sequence; this was deemed acceptable for the purposes of mutant gene rescue experiments.

For transformation, Chlamydomonas cells were grown to mid-log phase in liquid TAP media in air with the standard settings described above. Cells were then centrifuged at 1,000 *g* for 5 min and washed twice with 10 ml of MAX reagent (GeneArt MAX Efficiency Transformation Reagent for Algae, Invitrogen). Cells were resuspended to a concentration of 2 × 10^8^ cells per ml in MAX reagent. For each transformation, 60 µl of cells were combined with 5 µl of DNA that had been linearized in a standard restriction digest using the enzyme indicated in Supplementary Table 3 and their manufacturer protocol (New England Biolabs). This mixture was incubated at 4 °C for 5 min and then transferred to an electrocuvette (2-mm gap, Bulldog bio) that had been pre-cooled at 4 °C. Electroporation was done using a NEPAGENE NEPA21 electroporator (parameters: poring pulse, 250 volts, 8 ms pulse length, 50 ms pulse interval, 2 pulses, 40% decay rate, + polarity; transfer pulse, 20 volts, 50 ms pulse length, 50 ms pulse interval, 5 pulses, 40% decay rate, +/− polarity)^37^. Immediately following electroporation, the mixture was added to 8 ml TAP + 40 mM sucrose and incubated with agitation in the dark overnight. Cells were then pelleted at 600 *g* for 5 min and plated on TAP-agar solid plates with the appropriate antibiotics (that is, hygromycin or paromomycin). After 5 days of growth in dim light, plates were transferred to 100 µE light for 7–14 days until colonies appeared and were of a sufficient size for picking.

Constructs used to transform Arabidopsis are shown in Supplementary Table 4. The coding sequences of SAGA1, MITH1 and EPYC1 were codon optimized for expression in land plants and cloned into binary level 2 acceptor vectors using the Plant MoClo system^38^. The 35S cauliflower mosaic virus (CaMV) promoter, cassava vein mosaic virus (CsVMV) promoter and Arabidopsis RbcS3B promoter were used to drive expression. Preliminary experiments indicated that the Chlamydomonas MITH1 protein localizes to the cytosol of *Nicotiana benthamiana*; therefore, a chloroplast transit peptide (cTP) was added to MITH1 for expression in Arabidopsis. Level 2 vectors were transformed into *Agrobacterium tumefaciens* (AGL1) for stable insertion in Arabidopsis plants by floral dipping^39^.

To obtain the CrRBCS2;EPYC1;SAGA1;MITH1 lines, MITH1-mCerulean driven by either the 35S or RbcS3B promoter was expressed in the EP1S1-1 line^14^, consisting of SAGA1-mCherry and EPYC1-GFP expressed in the S2_Cr_ background^40^, in which the *1a3b* Rubisco small subunit mutant is complemented with Chlamydomonas Rubisco small subunit CrRBCS2. Transformants were selected on the basis of their kanamycin resistance. To obtain the CrRBCS2;EPYC1;MITH1 line, EPYC1-GFP was first expressed in the S2_Cr_ background; homozygous lines were identified in the T_3_ generation using a kanamycin resistance cassette. Then, MITH1-mCerulean driven by the RbcS3B promoter was transformed into the resulting line and transformants were selected using the pFAST-R selection cassette^41^.

### Transmission electron microscopy

Chlamydomonas samples for electron microscopy were prepared at room temperature and nutated in 1 ml volumes during chemical treatments and washes unless otherwise noted. After initial centrifugation for collection, all pelleting was done at 3,000 *g* for 1 min. Approximately 5 × 10^6^ cells were collected at 1,000 *g* for 5 min and fixed in 2.5% glutaraldehyde in TP medium for 1 h. After three 5-min washes in Milli-Q water, samples were treated with a freshly prepared solution of 1% OsO_4_, 1.5% K_3_Fe(CN)_3_ and 2 mM CaCl_2_. After four 5-min washes, samples were then serially dehydrated (5-min incubations in 50, 75, 95 and 100% ethanol, followed by two 10-min incubations in 100% acetonitrile). The samples were then suspended in 50% acetonitrile, 17.5% Quetol 651, 22.5% nonenyl succinic anhydride and 10% methyl-5-norbornene-2,3-dicarboxylic anhydride and left uncapped and stationary overnight in a fume hood to allow for evaporation of the acetonitrile. The samples were then embedded in epoxy resin containing 34% Quetol 651, 44% nonenyl succinic anhydride, 20% methyl-5-norbornene-2,3-dicarboxylic anhydride and 2% catalyst dimethylbenzylamine. The resin mixture was refreshed daily for 4 days. After the last resin refresh, pellets were resuspended in 300 µl of the resin mixture and centrifuged at 10,500 r.p.m. at 30 °C for 20 min in a swinging bucket rotor for microfuge tubes. They were then cured at 65 °C for 48 h. Afterwards, ultramicrotomy was done using a DiaTome diamond knife on a Leica UCT Ultramicrotome at the Imaging and Analysis Center, Princeton University, and imaging was done on a CM100 transmission electron microscope (Philips) at 80 kV or CM200 at 200 kV.

For Arabidopsis TEM, leaf samples were taken from 21-day-old plants and fixed with 4% (v/v) paraformaldehyde, 2% (v/v) glutaraldehyde and 0.1 M sodium cacodylate (pH 7.2). Leaf strips (1 mm wide) were vacuum infiltrated with fixative three times for 15 min, then rotated overnight at 4 °C. Samples were rinsed three times with PBS (pH 7.4), then dehydrated sequentially by vacuum infiltration with 50, 70, 80 and 90% ethanol (v/v) for 1 h each, then three times with 100% ethanol. Samples were infiltrated with increasing concentrations of LR White resin (30, 50 and 70% [w/v]) in ethanol overnight for each, then 100% resin three times for 1 h. The resin was polymerized in capsules at 50 °C overnight. Sections (1 μm thick) were cut on a Leica Ultracut ultramicrotome, stained with toluidine blue and viewed in a light microscope to select suitable areas for investigation. Ultrathin sections (60 nm thick) were cut from selected areas and mounted onto plastic-coated copper grids. Grids were stained in 2% (w/v) uranyl acetate, then viewed in a JEOL JEM-1400 Plus TEM (JEOL). Images were collected on a GATAN OneView camera (GATAN).

### Serial block-face scanning electron microscopy (SBF–SEM)

Leaf samples were harvested after 6 h light from 28-day-old plants grown in a 12-h day/night regime. Samples were chemically fixed, stained and embedded into Durcupan epoxy resin. All solutions were prepared as previously described^42^. Fixation and embedding was done in a Pelco BioWave Pro1 (Ted Pella) using an adaptation of a previously described protocol^43^ (see Supplementary Method ‘Epoxy resin embedding of arabidopsis leaf sections for light microscopy’). The resultant blocks were trimmed, glued on stubs, coated with a mixture of gold and palladium, and sectioned and scanned ~500 times using a scanning electron microscope (FEI Apreo VolumeScope, Thermo Fisher), operated at 1.18 kV. Each section removed 50 nm from the imaged block face. After image alignment, the structural features were marked out manually with Amira software (Thermo Fisher).

### Confocal microscopy

For several Chlamydomonas experiments including single-colour immunofluorescence imaging of CAH3 shown in Fig. 4e and Extended Data Fig. 8f, live imaging of CYN7-Venus shown in Fig. 3m–o and Extended Data Fig. 8e, live imaging of RBMP1-Venus shown in Fig. 4d, and all confocal imaging across Extended Data Figs. 7 and 8e–f, cells were imaged with a Leica TCS SP5 laser scanning confocal microscope using Leica LAS X software (Leica Microsystems) and a ×100 magnification/1.46 NA oil objective. Images were processed with FIJI software^44^. For the anti-CAH3 immunofluorescence experiment, excitation/emission wavelengths were 488 nm/515–535 nm. For live imaging, excitation/emission wavelengths were 514 nm/521–540 nm for Venus and 514 nm/645–725 nm for chlorophyll autofluorescence.

The RBCS1-Venus expressing strains shown in Fig. 4a were imaged using a Nikon A1R confocal laser scanning microscope (Nikon Instruments) with a ×100 magnification/1.45 NA oil immersion objective. After acquisition, Nikon NIS Elements software was used to optimize image quality using the Denoise.ai and Deconvolution tools. Excitation/emission wavelengths were 514 nm/522–555 nm for Venus and 514 nm/601–676 nm for chlorophyll autofluorescence.

All Arabidosis imaging as well as all Chlamydomonas imaging not specified above was performed with a Nikon CSU-W1 SoRa spinning disk system (Nikon Instruments) with a ×60 magnification/1.4 NA oil immersion objective. All acquisitions were magnified by an additional ×4 through SoRa magnification for a final magnification of ×240. After acquisition, Nikon NIS Elements software was used to optimize image quality using the Denoise.ai and Deconvolution tools. Excitation/emission wavelengths used for *Arabidopsis* imaging were 405 nm/430–480 nm for mCerulean, 488 nm/507–543 nm for GFP, 561 nm/579–631 nm for mCherry and 640 nm/669–741 nm for chlorophyll autofluorescence. Excitation/emission wavelengths used for Chlamydomonas live imaging were 514 nm/530–560 nm for Venus and 514 nm/669–741 nm for chlorophyll autofluorescence. Excitation/emission wavelengths used for two-colour immunofluorescence imaging were 488 nm/507–543 nm for Alexa Fluor 488 and 640 nm/669–741 nm for STAR RED.

### Immunofluorescence

Samples for immunofluorescence were prepared according to a published protocol^45^.

Briefly, cells were fixed through 20-min treatments with 4% (w/v) formaldehyde in PBS, followed by 100% methanol at −20 °C. Cells were then washed in PBS and blocked for 1 h in PBS containing 5% (w/v) bovine serum albumin, treated with primary antibody for 1 h and secondary antibody for another hour.

To visualize CAH3 localization in various strains, a rabbit polyclonal anti-CAH3 primary antibody (Agrisera) and Alexa Fluor 488-conjugated goat anti-rabbit secondary antibody (Thermo Fisher) were used.

For co-localization of CAH3 and MITH1, the *mith*;MITH1-Venus-3×FLAG strain was used. CAH3 was labelled with the rabbit polyclonal anti-CAH3 primary antibody and goat anti-rabbit Alexa Fluor 488-conjugated secondary antibody, and MITH1 was labelled using a mouse monoclonal anti-FLAG primary antibody (Sigma-Aldrich) and STAR RED-conjugated goat anti-mouse secondary antibody (Abberior).

For co-localization of CAH3 and SAGA1, the *saga1*;SAGA1-Venus-3×FLAG strain was used. CAH3 was labelled with the rabbit polyclonal anti-CAH3 primary antibody and goat anti-rabbit Alexa Fluor 488-conjugated secondary antibody, and SAGA1 was labelled using the mouse monoclonal anti-FLAG primary antibody and STAR RED-conjugated goat anti-mouse secondary antibody.

For co-localization of MITH1 and SAGA1, the *saga1*;SAGA1-Venus-3×FLAG strain was used. MITH1 was labelled with a rabbit anti-MITH1 primary antibody (Yenzyme) and goat anti-rabbit Alexa Fluor 488-conjugated secondary antibody, and SAGA1 was labelled using the mouse monoclonal anti-FLAG primary antibody and STAR RED-conjugated goat anti-mouse secondary antibody.

Antibody dilutions were 1:500 in PBST for all primary and secondary antibodies except for anti-CAH3, which was diluted 1:200 following supplier recommendation.

### Arabidopsis image processing and bleedthrough attenuation

Despite our best efforts to control bleedthrough by careful choice of filters and excitation wavelengths, in our microscopy setup for imaging Arabidopsis, bleedthrough from the EPYC1-GFP channel into the MITH1-mCerulean channel caused a non-negligible amount of false mCerulean signal (Extended Data Fig. 3a), which was particularly problematic considering the high expression levels of EPYC1-GFP and relatively low expression levels of MITH1-mCerulean. We opted to computationally attenuate the EPYC1-GFP bleedthrough signal from images presented in Fig. 2a–e and Extended Data Fig. 4c to provide the reader with a more-accurate representation of the localization of MITH1.

The procedure for bleedthrough attenuation is based on subtracting a portion of the signal measured in the GFP channel from the signal measured in the mCerulean channel. How much of the GFP signal to subtract in this procedure was set by a factor that we refer to as the ‘bleedthrough coefficient’. To determine the bleedthrough coefficient, we used images of condensates in the CrRBCS2;EPYC1-GFP line (lacking MITH1-mCerulean and therefore lacking a true mCerulean signal). To allow the bleedthrough coefficient to be approximated via a linear regression that passes through the origin (that is, when GFP signal is 0, bleedthrough in the mCerulean channel is 0), we first subtracted out a constant value across the entire image in each channel to account for the average ‘background’ signal due to factors other than GFP signal in that channel. This background signal includes noise inherent to the camera and sample autofluorescence. For instance, fluorescence not attributable to a fluorescent protein can be seen to varying degrees in the mCerulean channel of the CrRBCS2;EPYC1 line, apparently due to autofluorescence from the leaf sample itself (Extended Data Fig. 3a). By analysing images of the CrRBCS2;EPYC1 line, we empirically estimated a background signal of 200 intensity units in the mCerulean channel and 1,500 units in the GFP channel. These background values were subtracted from the corresponding channels for all images in a test set of CrRBCS2;EPYC1 images. A circular region of pixels was then sampled from each condensate, and the mCerulean intensity vs the GFP intensity was plotted for each such region as a data point in the regression. The slope of the regression (measured as 0.0134) was interpreted as the bleedthrough coefficient (Extended Data Fig. 3f).

In Extended Data Fig. 3b–e, we illustrate the bleedthrough attenuation procedure for a set of CrRBCS2;EPYC1-GFP;MITH1-mCerulean images. We first subtracted the constant background signal from the GFP and mCerulean channel images as described for the determination of the bleedthrough coefficient above (Extended Data Fig. 3c). We then created a copy of the GFP channel image and multiplied the signal intensity by the bleedthrough coefficient to match the expected intensity of the mCerulean bleedthrough. This rescaled GFP image was used as a mask on the mCerulean channel, and the intensity of this mask was subtracted from the mCerulean channel image on a pixel-by-pixel basis (Extended Data Fig. 3g) using the ND Images Arithmetics tool available on the Nikon NIS Elements software. This generated a new mCerulean channel image in which the signal corresponding to MITH1-mCerulean is conserved while the signal corresponding to bleedthrough is attenuated (Extended Data Fig. 3d). The chlorophyll and mCherry channels were denoised and deconvolved using NIS Elements software as described earlier. Since the EPYC1-GFP and MITH1-mCerulean channels were processed as described above, they were not deconvolved so as to avoid the possible introduction of artefacts (Extended Data Fig. 3e).

Scaling of the MITH1-mCerulean and SAGA1-mCherry channels is universal across Fig. 2a–d and across Extended Data Fig. 4c. This allowed for visual intensity to serve as a more direct indicator of protein levels to allow for comparison between strains. Scaling of the EPYC1-GFP and chlorophyll channels across Fig. 2a–d and Extended Data Fig. 4c was performed on an image-by-image basis such that maximum pixel intensity in the image determined the saturation point. This allowed consistency in the visual intensity of EPYC1 and chlorophyll regardless of variation between samples.

### Analysis of RBCS1-Venus puncta

To determine the number of condensates in each cell, Nikon NIS Elements software was used to threshold images on the basis of Venus signal intensity, disregarding parts of the image with no or very low intensity while representing continuous clusters of high-intensity pixels as binary objects, each representing a distinct RBCS1 condensate. Object identity was conserved over multiple *z*-slices, meaning that each was correctly identified as a single condensate even if it appeared across numerous slices.

Nikon NIS Elements software was used to measure the size of each condensate. For each condensate, which was detected and turned into a binary object as described above, the cross-sectional area was measured at each *z*-slice it appeared in, and the largest one was reported as the representative area of the condensate, as condensates are approximately spherical. This provided a reliable way of comparing condensate size in lieu of measuring volume, which is complicated by limits to *z*-resolution. To calculate the proportion of RBCS1 signal contained in the largest condensate of a given cell, a ratio was taken between the largest condensate area in the cell and the sum of all condensate areas in the cell.

Data tabulation, statistical analysis and data visualization were each performed using the GraphPad Prism 9 software. A two-tailed Mann–Whitney *U* test was used to test for significance of differences between strains when comparing the proportion of Rubisco fluorescence in the largest condensate of each cell.

### Cell fractionation

Wild-type Chlamydomonas was grown in TAP medium at ambient CO_2_ until they reached a density of 2 × 10^6^ cells per ml. Cells were centrifuged for 5 min at 1,000 × *g* and the pellet was weighed and resuspended in 2× volume of lysis buffer (50 mM HEPES, 10 mM KOAc, 2 mM Mg(OAC)_2_, 1 mM CaCl_2_ pH 7.0 + protease inhibitors). Cells were sonicated in a probe tip sonicator (Qsonica) on ice for 5 min, a 3-s pulse and 6-s rest at 60% amplitude. The lysate was centrifuged at 2,000 *g* for 10 min to remove intact cells, and 50 μl of supernatant was collected as the whole cell lysate (input) sample. The rest of the supernatant was divided in two; to half, *n*-dodecyl-*ß*-d-maltoside (DDM) was added to a final concentration of 1% and the mixture allowed to solubilize while nutating for 20 min at 4 °C. Samples were centrifuged at 18,000 × *g* for 30 min and pellet and supernatant were collected and analysed via western blotting.

### Western blot

For Chlamydomonas experiments, the PVDF membrane (Millipore) was blocked for 1 h in 5% milk in TBST (Tris buffered saline with 0.1% Tween-20), then incubated with primary antibody for 1 h. After three 10 min washes in TBST, the blot was treated with secondary antibody for 1 h and washed again 3× for 10 min. The secondary antibody was diluted 1:10,000. Anti-SAGA1 (ref. ^11^)(Yenzyme), anti-MITH1 (Yenzyme) and anti-ferredoxin (Agrisera) were diluted 1:1,000. Anti-RBMP1 (gift from L. Mackinder) and anti-CAH3 (rabbit polyclonal, Agrisera) were diluted 1:2,000. Anti-PsbC (Agrisera) was diluted 1:3,000 and anti-tubulin (mouse monoclonal, Sigma-Aldrich) was diluted 1:4,000. Blots were labelled using an enhanced chemiluminescence system (Advansta WesternBright ECL kit) and imaged on an iBright Imaging System (Thermo Fisher).

Protein analyses for Arabidopsis were done as follows. Soluble protein was extracted from frozen leaf material of 21-day-old plants in protein extraction buffer (20 mM Tris-HCl pH 7.5 with 5 mM MgCl_2_, 300 mM NaCl, 5 mM dithiothreitol, 1% Triton X-100 and cOmplete Mini EDTA-free Protease Inhibitor Cocktail (Roche)). Samples were heated at 80 °C for 15 min with 1× Bolt LDS sample buffer (Thermo Fisher) and 200 mM dithiothreitol. Extracts were centrifuged and the supernatants subjected to SDS–PAGE on a 12% (w/v) polyacrylamide gel and transferred to a nitrocellulose membrane. Membranes were probed with rabbit serum raised against SAGA1 (ref. ^13^) (1:1,000) or EPYC1 (ref. ^3^) (1:2,000), or with mouse anti-actin (Agrisera, AS21 4615) or mouse anti-GFP (Santa Cruz, sc-9996), followed by HRP-linked goat anti-rabbit IgG (Abcam) at 1:10,000 dilution, or rabbit anti-mouse IgG (Agrisera) at 1:10,000 dilution. Membranes were visualized using Pierce ECL Western Blotting Substrate (Life Technologies).

### Protein immunoprecipitation and mass spectrometry

Immunoprecipitation and mass spectrometry of Venus-3×FLAG-MITH1, Venus-3×FLAG-SAGA1 or Venus-3×FLAG were performed as described previously^23^ with the following differences: a 40 cm chromatography column was used, the column temperature was set to 45 °C and a 2-h gradient method with 300 nl min^−1^ flow was used.

Cells were grown in 1 l bottles of TP medium with air bubbling and constant stirring at 210 r.p.m. under 150 μmol photons m^−2^ s^−1^ of light until the cell density reached ∼2–4 × 10^6^ cells per ml. Cells were collected by centrifugation at 3,000 *g* for 4 min in an Avanti J-26X centrifuge with an 8.1000 rotor (Beckman) at 4 °C. The pellets were washed in 35 ml ice-cold washing buffer (25 mM HEPES, 25 mM KOAc, 1 mM Mg(OAc)_2_, 0.5 mM CaCl_2_, 100 mM sorbitol, 1 mM NaF, 0.3 mM Na_3_VO_4_ and cOmplete EDTA-free protease inhibitor (1 tablet per 500 ml)) and then resuspended in a 1:1 (v/w) ratio of ice-cold 2×IP buffer (50 mM HEPES, 50 mM KOAc, 2 mM Mg(OAc)_2_, 1 mM Cacl_2_, 200 mM sorbitol, 1 mM NaF, 0.3 mM Na_3_VO_4_ and cOmplete EDTA-free protease inhibitor (1 tablet per 50 ml)). Cell slurry (3 ml) was immediately added to liquid nitrogen to form small popcorn pellets which were stored at −80 °C until needed. Cells were lysed by cryogenic grinding using a Cryomill (Retsch) at a frequency of 25 oscillations per second for 20 min. The ground powder was defrosted on ice for 45 min and dounced 25 times on ice with a Kontes Duall #22 homogenizer (Kimble). Homogenized cells (1 ml) of each sample were used for the following processes. Membrane proteins were solubilized by incrementally adding an equal volume of ice-cold 1×IP buffer plus 2% digitonin (RPI), followed by an incubation of 45 min with nutation at 4 °C. The cell debris were removed by spinning at 12,700 *g* for 30 min at 4 °C. The supernatant was then mixed with 50 μl anti 3×FLAG magnetic beads (Sigma) which had been previously washed sequentially with 1×IP buffer 3 times and 1×IP buffer plus 0.1% digitonin 2 times. The mixture was incubated with nutation at 4 °C for 1.5 h, followed by the removal of supernatant. The beads were washed 4 times with 1×IP buffer plus 0.1% digitonin, followed by a 30-min competitive elution with 45 μl of 1×IP buffer plus 0.25% digitonin and 2 μg μl^−1^ 3×FLAG peptide (Sigma-Aldrich). After elution, 30 μl protein samples were mixed with 9.75 μl 4×SDS–PAGE buffer (BioRad) containing 100 mM dithiothreitol (Sigma-Aldrich), followed by denaturation by heating at 70 °C for 10 min. Then, 30 μl of denatured protein sample was loaded into a well of a 4–15% Criterion TGX Precast Midi Protein Gel (BioRad) for electrophoresis at 50 V for 40 min until the protein front moved ∼2.5 cm. Gel slices (∼2.0 cm) containing target proteins with molecular weight ≥10 kDa (to exclude the 3×FLAG peptide) were excised and stored at 4 °C until processing for in-gel digestion.

In-gel digestion of protein bands using trypsin was performed and the samples were dried completely in a SpeedVac and resuspended with 20 μl of 0.1% formic acid pH 3 in water. A volume of 2 μl (∼360 ng) was injected per run using an Easy-nLC 1,200 UPLC system. Samples were loaded directly onto a 40-cm-long, 75-μm-inner-diameter nanocapillary column packed with 1.9 μm C18-AQ resin (Dr. Maisch) mated to a metal emitter in line with an Orbitrap Fusion Lumos (Thermo Scientific). The column temperature was set at 45 °C and a 2-h gradient method with 300 nl min^−1^ flow was used. The mass spectrometer was operated in data-dependent mode with a 120,000 resolution MS1 scan (positive mode, profile data type, AGC gain of 4 × 10^5^, maximum injection time of 54 s and mass range of 375–1,500 *m*/*z*) in the Orbitrap, followed by HCD fragmentation in ion trap with 35% collision energy. A dynamic exclusion list was invoked to exclude previously sequenced peptides for 60 s, and a maximum cycle time of 3 s was used. Peptides were isolated for fragmentation using the quadrupole (1.2 *m*/*z* isolation window). The ion trap was operated in rapid mode.

### Spot tests

Cells were grown to mid-log phase in liquid TAP medium. They were then pelleted at 1,000 *g* for 5 min and resuspended to a concentration of 6 × 10^5^ cells per ml in TP medium. A volume of 10 µl of each sample was spotted onto TP-agar plates. Once the spots were dry, plates were then placed under 100 µmol photons m^−2^ s^−1^ of light in variable CO_2_ levels, which included 40 parts per million (ppm) CO_2_, ambient CO_2_ (~415 ppm) or 3% CO_2_ for 7 days before imaging on a Phenobooth imager. For the 40 ppm condition, plates were first kept in ambient air for the first 24 h before being placed in a 40 ppm chamber for the remaining 6 days.

### Statistics and reproducibility

Unless otherwise stated here, each microscopy image is representative of three or more biological repeats. One biological repeat was performed for the SBF–SEM experiment depicted in Fig. 2j and in Supplementary Videos 1–5. The results of this experiment are consistent with those produced using other imaging methods. Representative images from two biological repeats are shown for the TEM images of the CrRBCS2;EPYC1;MITH1 Arabidopsis line presented in Fig. 2i and Extended Data Fig. 4a, the co-localization of SAGA1-Venus and CAH3 shown in Fig. 3i, the *CrRBCS2;EPYC1-GFP* images shown in Extended Data Fig. 3a, the *mith1;saga1* double mutant TEM images shown in Extended Data Fig. 6b and the wild-type cell shown in Extended Data Fig. 8a.

Four biological repeats were performed for the CAH3 immunofluorescence experiment presented in Fig. 4e, although two replicates were imaged on a Leica TCS SP5 laser scanning confocal microscope (Leica Microsystems) and the other two on a Nikon CSU-W1 SoRa spinning disk system (Nikon Instruments). For consistency, data from the two replicates imaged with the Nikon CSU-W1 SoRa spinning disk system (Nikon Instruments) microscope were used to generate the graph in Fig. 4f. Exact *P* values for the graph in Fig. 4f are as follows: WT vs *mith1*, *P* = 0.0195; WT vs *saga1*, *P* = 1.15 × 10^−6^; WT vs *cah3*, *P* = 3.82 × 10^−8^.

### Reporting summary

Further information on research design is available in the Nature Portfolio Reporting Summary linked to this article.

## Supplementary information

Supplementary Information Supplementary Tables 1–4 and Figs. 1–4.

Reporting Summary

Supplementary Table 5 Immunoprecipitation–mass spectrometry (IP–MS) dataset. The full dataset used to produce Fig. 3q,r is provided in a spreadsheet. MITH1-Venus-3×FLAG or SAGA1-Venus-3×FLAG were used as baits, and the results from two replicates were averaged. Venus-3×FLAG was used as a control bait to test for non-specific interactions.

Supplementary Video 1 Serial block face scanning electron microscopy (SBF–SEM) of leaves from *CrRBCS2;EPYC1-GFP;SAGA1-mCherry;MITH1-mCerulean* *Arabidopsis* where MITH1 is expressed under a 35S constitutive promoter.

Supplementary Video 2 SBF–SEM of leaves from *CrRBCS2;EPYC1-GFP;SAGA1-mCherry;MITH1-mCerulean* *Arabidopsis* where MITH1 is expressed under a 35S constitutive promoter.

Supplementary Video 3 SBF–SEM of leaves from *CrRBCS2;EPYC1-GFP;SAGA1-mCherry;MITH1-mCerulean* *Arabidopsis* where MITH1 is expressed under the weaker RbcS3B promoter.

Supplementary Video 4 SBF–SEM of leaves from a control *CrRBCS2;EPYC1-GFP* *Arabidopsis* line.

Supplementary Video 5 SBF–SEM of leaves from a control *CrRBCS2;EPYC1-GFP* *Arabidopsis* line.

## Data Availability

The *Chlamydomonass reinhardtii* v.5.6 genome (Phytozome Accession ID: ABCN02000000) was referenced in this study. All data generated or analysed during this study are included in this published Article and its supplementary information files.
