# Supplementary material for: SAGA1 and MITH1 produce matrix-traversing membranes in the CO2-fixing pyrenoid
Source: Nat Plants. 2024 Nov 15;10(12):2038–51. doi: 10.1038/s41477-024-01847-0 (PMC11649565; doi:10.1038/s41477-024-01847-0)
Supplement: Supplementary file 1 — Supplementary Tables 1–4 and Figs. 1–4. [file 41477_2024_1847_MOESM1_ESM.pdf]

nature plants

Supplementary information https://doi.org/10.1038/s41477-024-01847-0

SAGA1 and MITH1 produce matrix-traversing membranes in the CO_2-fixing pyrenoid

In the format provided by the authors and unedited

Title: SAGA1 and MITH1 produce matrix-traversing membranes in the CO_2-fixing pyrenoid

Authors: Jessica H. Hennacy[1], Nicky Atkinson[2,8], Angelo Kayser-Browne[1,8], Sabrina L. Ergun[1,3,8], Eric Franklin[1], Lianyong Wang[1], Simona Eicke[4], Yana Kazachkova[1], Moshe Kafri[1], Friedrich Fauser[1], Josep Vilarrasa-Blasi[5], Robert E. Jinkerson[6,7], Samuel C. Zeeman[4], Alistair J. McCormick[2*], Martin C. Jonikas[1,3*]

Affiliations:

[1]Department of Molecular Biology, Princeton University; Princeton, NJ, 08544, US.

[2]Institute of Molecular Plant Sciences, School of Biological Sciences, University of Edinburgh, Edinburgh, Scotland, EH9 3BF, UK.

[3]Howard Hughes Medical Institute, Princeton University, Princeton, NJ, 08544, US.

[4]Department of Biology, ETH Zurich, Zurich 8092, Switzerland

[5]Department of Plant Biology, Carnegie Institution for Science, Stanford, CA, USA

[6]Department of Botany and Plant Sciences, University of California, Riverside, CA, USA.

[7]Department of Chemical and Environmental Engineering, University of California, Riverside, CA, USA.

[8]These authors contributed equally.

[*]Corresponding authors. Email: alistair.mccormick@ed.ac.uk and mjonikas@princeton.edu

Supplementary Tables

Chlamydomonas Resource Center ID	Strain Description	Mating type (mt)	Source	Antibiotic resistance
CC-4533	*CMJ030 (CC-4533)*	Minus (mt-)	Wildtype and parent strain to CLiP library[35]	none
LMJ.RY0402.133670	*mith1*	mt-	CLiP library LMJ.RY0402.133670[36]	Paromomycin
CC-5938	*mith1;MITH1-Venus-3xFLAG*	mt-	Transformation of *mith1* mutant with linearized *MITH1-Venus-3xFLAG pRAM118* plasmid	Paromomycin, hygromycin
CC-5420	*saga1*	mt-	Previously published[16]	Paromomycin
CC-5939	*CMJ030; CYN7-Venus-3xFLAG*	mt-	Transformation of CMJ030 with linearized CYN7-Venus-3xFLAG pLM005 plasmid	Paromomycin
CC-5940	*mith1;CYN7-Venus-3xFLAG*	mt-	Transformation of *mith1* with linearized CYN7-Venus-3xFLAG pLM005 plasmid	Paromomycin, Hygromycin
CC-5941	*saga1;CYN7-Venus-3xFLAG*	mt-	Transformation of *saga1* with with linearized CYN7-Venus-3xFLAG pLM005 plasmid	Paromomycin, Hygromycin
CC-5617	*CMJ030;RBMP1-Venus-3xFLAG*	mt-	Previously published[24]	Paromomycin
CC-5155	*CC-5155*	mt+	Wildtype strain made through cross between CMJ030 mt-and CC-125 mt+ followed by 5 times backcrossing to CMJ030; Chlamydomonas Resource Center CC-5155	none
CC-5942	*CMJ030; RBCS1-Venus-3xFLAG*	Plus (mt+)	Crossing of the previously published[1] CMJ030;RBCS1-Venus-3xFLAG mt- strain to the wildtype CC1690 mt+ strain	Paromomycin
	Rubisco mutant D23A/E24A11	mt-	Previously published[10]	none
CC-5943	*saga1;sta6*	Unknown	Crossing of *saga1* mt- with *sta6* mt+	Paromomycin
CC-5944	*saga1;sta6;SAGA1-Venus-3xFLAG*	Unknown	Transformation of *saga1;sta6* double mutant with linearized SAGA1-Venus-3xFLAG pRAM118 plasmid	Paromomycin, Hygromycin
CC-5374	*sta6*	mt+	Chlamydomonas Resource Center C5374	none
CC-5422	*saga1;SAGA1-Venus-3xFLAG*	mt-	Previously published[16]	Paromomycin, Hygromycin
CC-5945	*saga1; RBCS1-Venus-3xFLAG*	Unknown	Crossing of *saga1* mt- with CMJ030; RBCS1-Venus-3xFLAG mt+	Paromomycin
CC-5946	*mith1; RBCS1-Venus-3xFLAG*	Unknown	Crossing of *mith1* mt- with CMJ030; RBCS1-Venus-3xFLAG mt+	Paromomycin
CC-5947	*saga1 mt+*	mt+	Crossing of *saga1* mt- with CC-5155 mt+	Paromomycin
CC-5948	*mith1;saga1*	Unknown	Crossing of *mith1* mt- with *saga1* mt+	Paromomycin
CC-5949	*mith1;saga1;MITH1-Venus-3xFLAG*	Unknown	Transformation of *mith1;saga1* with linearized MITH1-Venus-3xFLAG pRAM118 plasmid	Paromomycin, Hygromycin
CC-5950	*mith1;saga1;SAGA1-Venus-3xFLAG*	Unknown	Transformation of *mith1;saga1* with linearized SAGA1-Venus-3xFLAG pRAM118 plasmid	Paromomycin, Hygromycin
CC-5951	*saga1;RBMP1-Venus-3xFLAG*	mt-	Co-transformation of *saga1* with linearized RBMP1-Venus-3xFLAG pLM164 plasmid and Hygromycin resistance gene from pRAM118	Paromomycin, Hygromycin
	sta6;RBCS1-Venus-3xFLAG	mt+	Transformation of *sta6* with RBCS1-Venus-3xFLAG pRAM118 plasmid	Hygromycin
	saga1;sta6;RBCS1-Venus-3xFLAG	unknown	Transformation of *saga1;sta6* with RBCS1-Venus-3xFLAG pRAM118 plasmid	Paromomycin, Hygromycin
LMJ.RY0402.174216	*cah3*	mt-	CLiP library mutant LMJ.RY0402.174216[36]	Paromomycin

Supplementary Table 1 | List of Chlamydomonas strains used in this study and their sources. Unless otherwise noted, the wild-type CMJ030 (CC-4533)[35] was the background for all strains. Mutants from the CLiP library were produced using insertional mutagenesis with a paromomycin resistance gene[36]. New strains produced for this study were made through crossing and transformation, as described in the table.

Arabidopsis Line	Source	Plasmids Used
CrRbcS2	Previously published[37]	CrRbcS2
CrRbcS2;EPYC1	Previously published[12]	CrRbcS2, EPYC1L2.188
CrRbcS2;EPYC1;SAGA1	Previously published[17]	CrRbcS2, EPYC1;SAGA1L2.150
CrRbcS2;EPYC1;MITH1	This manuscript	CrRbcS2, EPYC1L2.188, MITH1L2.259
CrRbcs2;EPYC1;SAGA1;MITH1 (MITH1 constitutive promoter)	This manuscript	CrRbcS2, EPYC1;SAGA1L2.150, MITH1L2.213
CrRbcs2;EPYC1;SAGA1;MITH1 (MITH1 RbcS3B promoter)	This manuscript	CrRbcS2, EPYC1;SAGA1L2.150, MITH1L2.214

Supplementary Table 2 | List of Arabidopsis lines used in this study. See Supplementary Table 4 for details about the plasmids used.

Plasmid	Source	Chlamydomonas antibiotic resistance	*E. Coli* antibiotic resistance	Restriction enzymes
***MITH1-Venus-3xFLAG* pRAM118**	InFusion (Takara Bio) cloning of PCR-amplified *MITH1* into HpaI-linearized PRAM118 vector in-frame with *Venus-3xFLAG*	Hygromycin	Ampicillin	EcoRV
***CYN7-Venus-3xFLAG* pLM005**	Previously published[26]	Paromomycin	Ampicillin	BsaI
***SAGA1-Venus-3xFLAG* pRAM118**	Previously published[16]	Hygromycin	Ampicillin	NdeI
***RBMP1-Venus-3xFLAG* pLM164**	Previously published	Paromomycin	Ampicillin	SpeI
***RBCS1-Venus-3xFLAG* pRAM118**	Gibson assembly (NEB) cloning of PCR-amplified *RBCS1* into HpaI-linearized pRAM118 vector in-frame with *Venus-3xFLAG*	Hygromycin	Ampicillin	Not1

Supplementary Table 3 | Plasmids used for transformation into Chlamydomonas. The indicated restriction enzyme was used to linearize the plasmid before transformation.

Plasmid Name	Promoter	cTP	Gene	Tag	Terminator	Selection
**CrRbcS2**	AtRbcS1A	RbcS1a cTP	Mature Chlamydomonas RbcS2	none	35S	BASTA
**EPYC1L2.188**	CsVMV	RbcS1a cTP	EPYC1	tGFP	HSP and Nos	Kanamycin
**EPYC1;SAGA1L2.150**	Gene 1: 35S<br>Gene 2: CsVMV	Gene 1: none<br>Gene 2: RbcS1a cTP	Gene 1: SAGA1<br>Gene 2: EPYC1	Gene 1: mCherry<br>Gene 2: tGFP	Gene 1: 35S and Nos<br>Gene 2: HSP and Nos	pFast red seeds
**MITH1L2.259**	RbcS3B	RbcS1a cTP	MITH1	mCerulean	HSP and Nos	pFast red seeds
**MITH1L2.213**	35S	RbcS1a cTP	MITH1	mCerulean	35S and Nos	Kanamycin
**MITH1L2.214**	RbcS3B	RbcS1a cTP	MITH1	mCerulean	35S and Nos	Kanamycin

Supplementary Table 4 | Plasmids used for transformation into Arabidopsis.

Supplementary Table 5 | Immunoprecipitation-mass spectrometry (IP-MS) dataset. The full dataset used to produce Fig. 3q, r is provided in a spreadsheet. MITH1-Venus-3xFLAG or SAGA1-Venus-3xFLAG were used as baits, and the results from two replicates were averaged. Venus-3xFLAG was used as a control bait to test for non-specific interactions.

Supplementary Videos

Supplementary Video 1 | Serial block face scanning electron microscopy (SBF-SEM) of leaves from *CrRBCS2;EPYC1-GFP;SAGA1-mCherry;MITH1-mCerulean* Arabidopsis where MITH1 is expressed under a 35S constitutive promoter.

Supplementary Video 2 | Serial block face scanning electron microscopy (SBF-SEM) of leaves from *CrRBCS2;EPYC1-GFP;SAGA1-mCherry;MITH1-mCerulean* Arabidopsis where MITH1 is expressed under a 35S constitutive promoter.

Supplementary Video 3 | Serial block face scanning electron microscopy (SBF-SEM) of leaves from *CrRBCS2;EPYC1-GFP;SAGA1-mCherry;MITH1-mCerulean* Arabidopsis where MITH1 is expressed under the weaker RbcS3B promoter.

Supplementary Video 4 | Serial block face scanning electron microscopy (SBF-SEM) of leaves from a control *CrRBCS2;EPYC1-GFP* Arabidopsis line.

Supplementary Video 5 | Serial block face scanning electron microscopy (SBF-SEM) of leaves from a control *CrRBCS2;EPYC1-GFP* Arabidopsis line.

Supplementary Figures

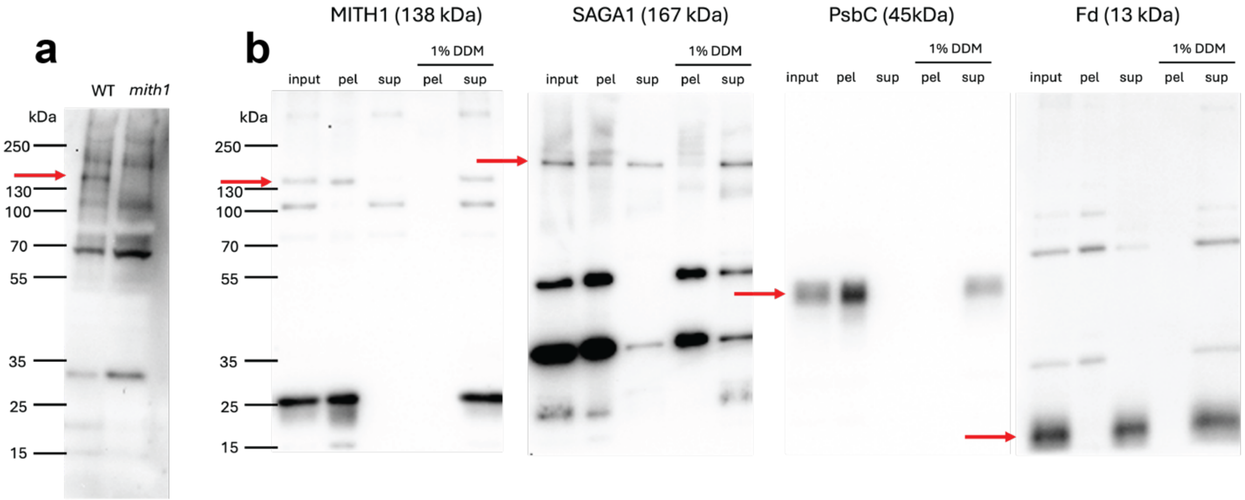

Supplementary Fig. 1 | Full gels of cell fractionation and western blot experiments. a, A blot verifying the ability of the anti-MITH1 antibody to bind MITH. **b,** Full blots corresponding to the cell fractionation experiment in Fig. 3k. Samples were evenly split, and each antibody was probed individually on a membrane. In each gel, an arrow points to the band corresponding to the protein of interest.

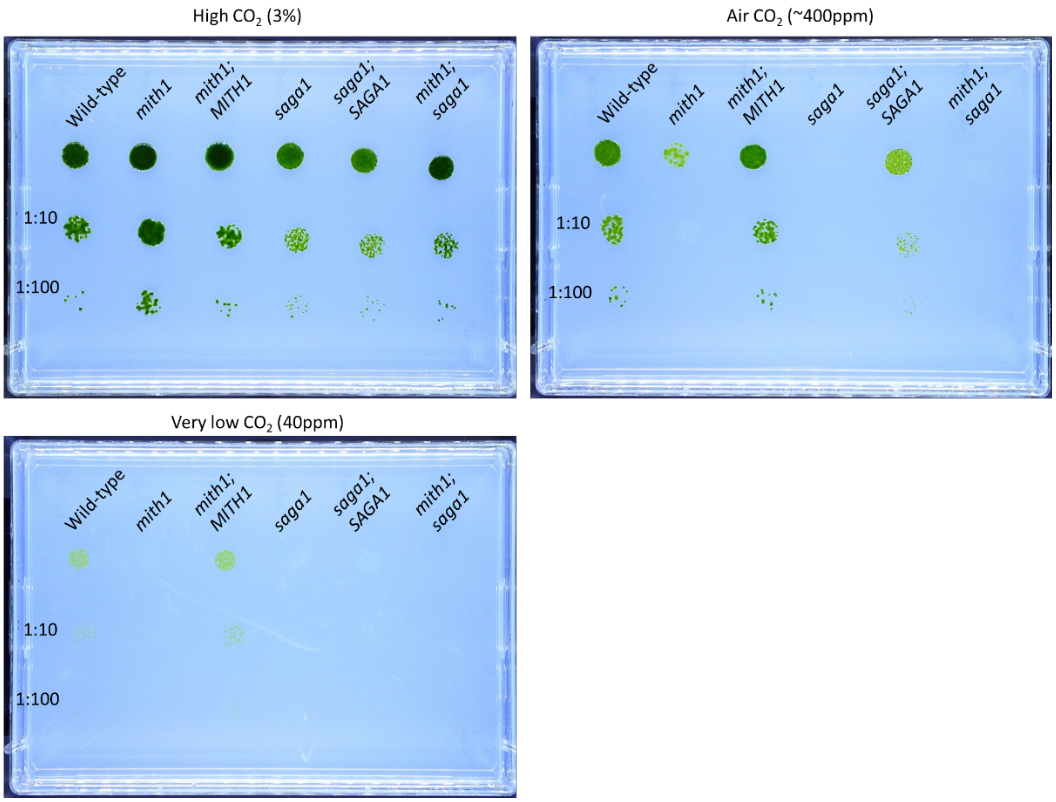

Supplementary Fig. 2 | Spot test depicting growth at varying levels of CO_2 and 100 µmol photons $m^{-2}{\cdot}s^{-1}$ light levels. Full plates corresponding to Fig. 3l are shown here. Spots that were diluted 1:10 and 1:100 are also depicted.

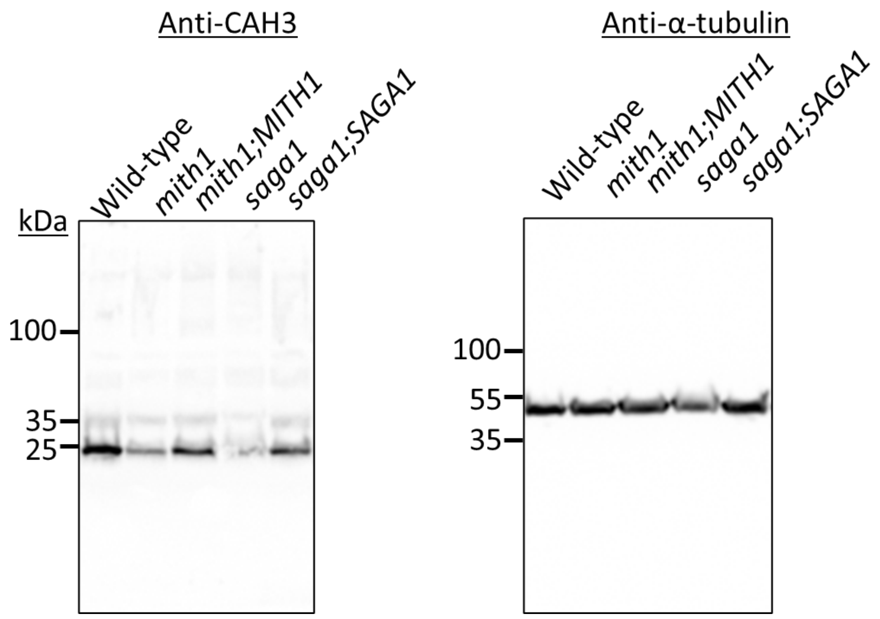

Supplementary Fig. 3 | Anti-CAH3 western blot performed on whole cell lysates. Full blots corresponding to Fig. 4g are shown. The same blot was stripped and probed with anti-α-tubulin as a loading control.

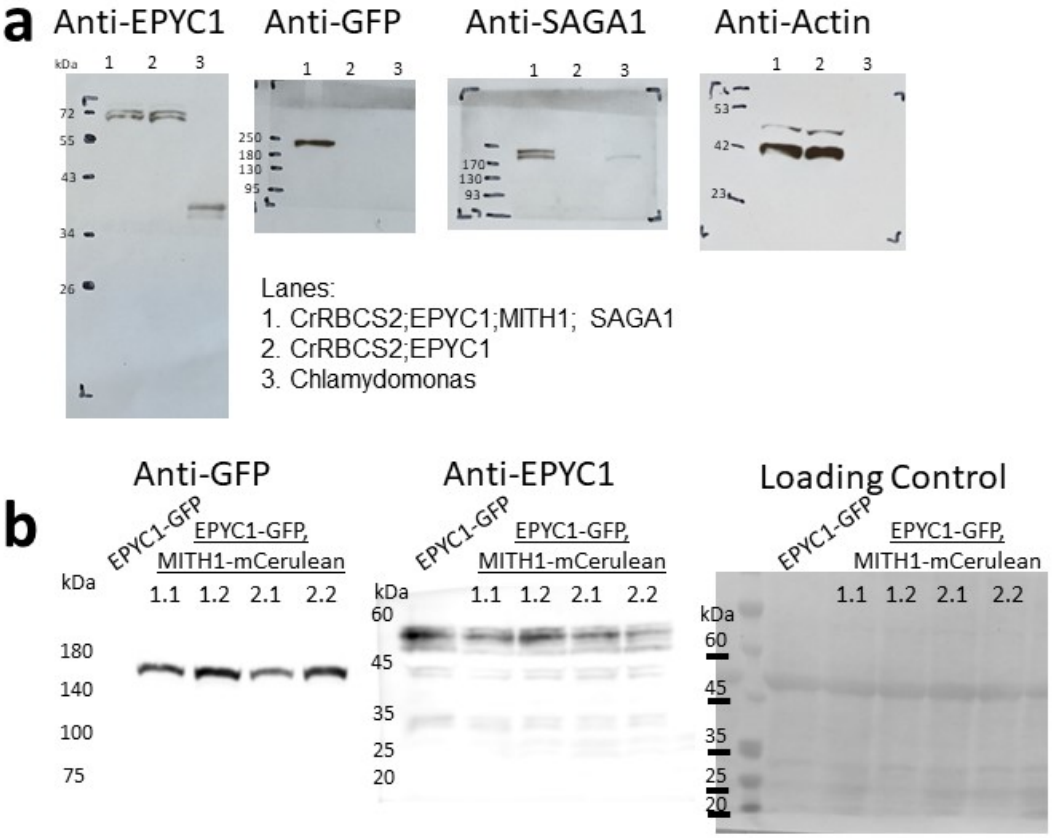

Supplementary Fig. 4 | MITH1-mCerulean and SAGA1-mCherry were heterologously expressed in an *Arabidopsis* line containing CrRBCS1 and EPYC1-GFP. Full blots corresponding to Extended Data Fig. 2 are shown. **a,** Western blots were performed on soluble protein extracts run through a denaturing SDS-PAGE gel to probe for **a,** proteins expressed in the Arabidopsis *CrRBCS2;EPYC1-GFP;MITH1-mCerulean;SAGA1-mCherry* line (lane 1) and *CrRBCS2;EPYC1-GFP* line (lane 2) as compared to the native proteins from Chlamydomonas (lane 3) and **b,** proteins expressed in the *CrRBCS2;EPYC1-GFP* line (lane 1) compared to C*rRBCS2;EPYC1-GFP;MITH1-mCerulean* plants (lanes 2-5). The anti-GFP antibody was used

to detect mCerulean. In (a), Actin is used as a loading control for the Arabidopsis samples. In (b), a Ponceau stain is used to detect total protein as a loading control.
