# Supplementary material for: SAGA1 and MITH1 produce matrix-traversing membranes in the CO2-fixing pyrenoid
Source: Nat Plants. 2024 Nov 15;10(12):2038–51. doi: 10.1038/s41477-024-01847-0 (PMC11649565; doi:10.1038/s41477-024-01847-0)
Supplement: Supplementary file 2 — Reporting Summary [file 41477_2024_1847_MOESM2_ESM.pdf]

nature portfolio

Corresponding author(s): Martin C. Jonikas
Alistair J. McCormick

Last updated by author(s): 10/4/2024

Reporting Summary

Nature Portfolio wishes to improve the reproducibility of the work that we publish. This form provides structure for consistency and transparency in reporting. For further information on Nature Portfolio policies, see our Editorial Policies and the Editorial Policy Checklist.

Statistics

For all statistical analyses, confirm that the following items are present in the figure legend, table legend, main text, or Methods section.

n/a	Confirmed	
☐	☒	The exact sample size (*n*) for each experimental group/condition, given as a discrete number and unit of measurement
☐	☒	A statement on whether measurements were taken from distinct samples or whether the same sample was measured repeatedly
☐	☒	The statistical test(s) used AND whether they are one- or two-sided<br>*Only common tests should be described solely by name; describe more complex techniques in the Methods section.*
☒	☐	A description of all covariates tested
☐	☒	A description of any assumptions or corrections, such as tests of normality and adjustment for multiple comparisons
☒	☐	A full description of the statistical parameters including central tendency (e.g. means) or other basic estimates (e.g. regression coefficient) AND variation (e.g. standard deviation) or associated estimates of uncertainty (e.g. confidence intervals)
☐	☒	For null hypothesis testing, the test statistic (e.g. *F*, *t*, *r*) with confidence intervals, effect sizes, degrees of freedom and *P* value noted<br>*Give P values as exact values whenever suitable.*
☒	☐	For Bayesian analysis, information on the choice of priors and Markov chain Monte Carlo settings
☒	☐	For hierarchical and complex designs, identification of the appropriate level for tests and full reporting of outcomes
☒	☐	Estimates of effect sizes (e.g. Cohen's *d*, Pearson's *r*), indicating how they were calculated

Our web collection on statistics for biologists contains articles on many of the points above.

Software and code

Policy information about availability of computer code

Data collection	Nikon NIS Elements, Leica LAS X
Data analysis	FIJI, Leica LAS X, Nikon NIS Elements, GraphPad Prism 9, Amira

For manuscripts utilizing custom algorithms or software that are central to the research but not yet described in published literature, software must be made available to editors and reviewers. We strongly encourage code deposition in a community repository (e.g. GitHub). See the Nature Portfolio guidelines for submitting code & software for further information.

Data

Policy information about availability of data

All manuscripts must include a data availability statement. This statement should provide the following information, where applicable:

- Accession codes, unique identifiers, or web links for publicly available datasets
- A description of any restrictions on data availability
- For clinical datasets or third party data, please ensure that the statement adheres to our policy

The Chlamydomonass reinhardtii v5.6 genome (Phytozome Accession ID: ABCN02000000) was referenced in this study. All data generated or analysed during this study are included in this published article (and its supplementary information files).

Human research participants

Policy information about studies involving human research participants and Sex and Gender in Research.

Reporting on sex and gender	n/a
Population characteristics	n/a
Recruitment	n/a
Ethics oversight	n/a

Note that full information on the approval of the study protocol must also be provided in the manuscript.

Field-specific reporting

Please select the one below that is the best fit for your research. If you are not sure, read the appropriate sections before making your selection.

☒ Life sciences ☐ Behavioural & social sciences ☐ Ecological, evolutionary & environmental sciences

For a reference copy of the document with all sections, see nature.com/documents/nr-reporting-summary-flat.pdf

Life sciences study design

All studies must disclose on these points even when the disclosure is negative.

Sample size	For both electron microscopy and light microscopy, at least 40 cells of each sample were imaged to ensure that the data were representative. The sample size was chosen based on past experience and standards in the field. Sample size for imaging experiments was consistent with what has been accepted in the field. For example, sample size was consistent with what was collected for a chloroplast protein atlas described in Wang et al 2023 (doi: 10.1016/j.cell.2023.06.008.)
Data exclusions	Data was not excluded
Replication	Nearly all experiments were successfully replicated in triplicate. In rare cases where only two replicates were performed, this is indicated.
Randomization	Randomization was not used in this study. All samples (controls and experimental samples) were tested with the same methods in parallel.
Blinding	Blinding was not used in this study, as objective variables were measured (e.g., presence/absence of clear phenotypes, localizations of proteins), and appropriate controls used in all experiments. For imaging data, fields of cells were imaged in addition to individual zooms to ensure that the researcher was not selecting favorable cells to examine. Furthermore, consistent results were obtained across multiple, independent methods, reducing the impact of bias in any particular method.

Reporting for specific materials, systems and methods

We require information from authors about some types of materials, experimental systems and methods used in many studies. Here, indicate whether each material, system or method listed is relevant to your study. If you are not sure if a list item applies to your research, read the appropriate section before selecting a response.

Materials & experimental systems

n/a	Involved in the study	
☐	☒	Antibodies
☒	☐	Eukaryotic cell lines
☒	☐	Palaeontology and archaeology
☒	☐	Animals and other organisms
☒	☐	Clinical data
☒	☐	Dual use research of concern

Methods

n/a	Involved in the study	
☒	☐	ChIP-seq
☒	☐	Flow cytometry
☒	☐	MRI-based neuroimaging

Antibodies

Antibodies used	Monoclonal mouse anti-alpha tubulin: Sigma-Aldrich, T5168, clone B-5-1-2<br>Polyclonal rabbit anti-CAH3: Agrisera, AS11 1757

Polyclonal rabbit anti-SAGA1: Yenzyme, custom, #5555
Polyclonal rabbit anti-ferredoxin: Agrisera, ASO6 121
Polyclonal rabbit anti-MITH1: Yenzyme, custom, #7559
Monoclonal mouse anti-FLAG: Sigma-Aldrich, F1804, clone M2
Polyclonal goat anti-rabbit, Alexa Fluor 488-conjugated: ThermoFisher, A11034
Polyclonal goat anti-rabbit, HRP-conjugated: ThermoFisher, PI31466
Goat anti-mouse, STAR RED-conjugated: Abberior, STRED-1001

Validation

Anti-alpha tubulin: independent enhanced validation, as stated on manufacturer’s website, which includes testing for applications in Western Blotting. Manufacturer's website lists Chlamydomonas as species that antibody is reactive in, and we have observed that a <55 kDa protein band is recognized by this antibody in all our Chlamydomonas samples
Anti-CAH3: validated with Western blot in Chlamydomonas wildtype vs. cah3 mutant, supplied on manufacturer’s website. Validated for immunofluorescence of Chlamydomonas cells in Extended Data Fig. 5k
Anti-SAGA1: validated with Western blot in Chlamydomonas wildtype vs. saga1 mutant (Meyer 2020)
Anti-MITH1: validated with Western blot in Chlamydomonas wildtype vs. mith1 mutant (validating data collected for this manuscript is available in Extended Data Fig. 5k and Supplementary Figure 1a)
Anti-FLAG: purity tested with microfluidic gel capillary electrophoresis as written on manufacturer’s website. FLAG epitope is added as a tag to our proteins expressed in Chlamydomonas through molecular cloning. Validated for immunofluorescence of Chlamydomonas cells in Extended Data Fig. 5k. Anti-FLAG is used for pulling down FLAG-tagged proteins expressed in Chlamydomonas in doi: 10.1016/j.cell.2023.06.008
Anti-ferredoxin: manufacturer's website cites publication (DOI: 10.1074/jbc.M109.023622), which validates reactivity of antibody through Western blotting of Chlamydomonas reinhardtii chloroplast ferredoxins
